# *Brassica* Genus Seeds: A Review on Phytochemical Screening and Pharmacological Properties

**DOI:** 10.3390/molecules27186008

**Published:** 2022-09-15

**Authors:** Jawaher Ayadi, Mohamed Debouba, Rami Rahmani, Jalloul Bouajila

**Affiliations:** 1 Laboratoire de Recherche, Biodiversité, Molécule et Application, Institut Supérieur de Biologie Appliquée de Médenine, Université de Gabès, Zrig, Gabès 6072, Tunisia; 2 Laboratoire de Génie Chimique, Université de Toulouse, CNRS, INPT, UPS, F-31062 Toulouse, France

**Keywords:** *Brassica* plants, seeds, oilseeds, nutrients, bioactive phytochemicals, pharmacological activities, cancer, diabetes, COVID-19, toxicity

## Abstract

Traditionally, *Brassica* species are widely used in traditional medicine, human food, and animal feed. Recently, special attention has been dedicated to *Brassica* seeds as source of health-promoting phytochemicals. This review provides a summary of recent research on the *Brassica* seed phytochemistry, bioactivity, dietary importance, and toxicity by screening the major online scientific database sources and papers published in recent decades by Elsevier, Springer, and John Wiley. The search was conducted covering the period from January 1964 to July 2022. Phytochemically, polyphenols, glucosinolates, and their degradation products were the predominant secondary metabolites in seeds. Different extracts and their purified constituents from seeds of *Brassica* species have been found to possess a wide range of biological properties including antioxidant, anticancer, antimicrobial, anti-inflammatory, antidiabetic, and neuroprotective activities. These valuable functional properties of *Brassica* seeds are related to their richness in active compounds responsible for the prevention and treatment of various chronic diseases such as obesity, diabetes, cancer, and COVID-19. Currently, the potential properties of *Brassica* seeds and their components are the main focus of research, but their toxicity and health risks must also be accounted for.

## 1. Introduction

The *Brassicaceae* family, commonly called *crucifers*, stands out as one of the most frequently cultivated and consumed all over the world with around 338 genera and more than 3700 species [1]. In recent years, *cruciferous* vegetables have attracted increased attention as they represent an excellent source of nutrients (carbohydrates, lipids, proteins, vitamins, and minerals) and health-promoting phytochemicals (phenolics, flavonoids, and glucosinolates) responsible for the prevention and treatment of various diseases via several biological qualities, including anti-obesity, antioxidant, anticancer, antimicrobial, anti-inflammatory, and antidiabetic activities [1,2,3,4].

Among all the *Brassicaceae* family genera, the *Brassica* (B.) genus is the most known and the most important one [5]. It includes important vegetables, oilseed crops, and forage species, divided into six species. *Brassica nigra* L., *Brassica oleracea* L., and *Brassica rapa* L. are three diploid species, whereas *Brassica carinata*, *Brassica juncea* (L.), and *Brassica napus* L. are all amphidiploid [6]. These species are grown mostly in the northern hemisphere’s Mediterranean, temperate, and cold climates [7]. *B. oleracea*, the dominant vegetable species, includes a wide range of morphological variants such as broccoli (*B. oleracea* var. *italica*), cabbage (*B. oleracea* var. *capitata*), kohlrabi (*B. oleracea* var. *gongylodes*), cauliflower (*B. oleracea* var. *botrytis*), Brussels sprouts (*B. oleracea* var. *gemmifera*), and kale (*Brassica oleracea* var. *acephala*) [8]. *B. napus*, including oilseed rape or canola, is an allotetraploid oilseed crop derived from *B. oleracea* L. and *B. rapa* L. [9]. The botanical classifications and dietary nomenclature of the most cultivated and consumed *Brassica* species worldwide are listed in Table 1.

The *Brassica* genus constitutes a potential reservoir for food products with high economic and medicinal value in the world owing to the synergistic action of its bioactive compounds [6,11]. The phytochemical screening and the beneficial properties in the vegetative organs of various *Brassica* species have been well documented, such as *B. nigra* [12], *B. oleracea* [8,13], *B. rapa* [14], *B. carinata* [15], *B. juncea* [16,17], and *B. napus* [18,19].

*Brassica* species are mainly cultivated as vegetable crops, as fodder, and for their seeds as spices and oil sources. In particular, rapeseed oil is the world’s third-largest producer of edible oil, behind only soybeans and palm trees, accounting for 14% of global production [10,20]. Over the past few years, Nepal has been the leading producer of mustard seeds, with more than 32% of the worldwide global production in 2019, followed by Russia with 25% and Canada with 21%. The US, Germany, and France were the global highest importers of yellow and brown mustard seeds used as condiment and cooking oil. Notably, the FAOSTAT (Food and Agriculture Organization of the United Nations, 2021) data report that the UK (221,000 tons), Germany (202,000 tons), and Italy (190,000 tons) consumed the most prepared mustard worldwide in 2019 [21].

Recently, special emphasis has been placed on the seeds of *Brassica* vegetables, and several research studies conducted on phytochemical screening have revealed that *Brassica* seeds, like all other organs of the vegetative system, are a very rich source of nutrients (carbohydrates, fats, proteins, vitamins, and minerals), and they contain a broad spectrum of various bioactive secondary metabolites of medicinal value, mainly phenolic compounds, glucosinolates and carotenoids. This richness in nutritional and medicinal compounds offers *Brassica* seeds a strong bioactive potential, mainly antioxidant, antiproliferative, antimicrobial, antidiabetic, anti-inflammatory, and neuroprotective properties.

Considering the versatility of the *Brassica* genus, this review provides a comprehensive summary of research progress on the *Brassica* species seed traditional and agronomic uses, phytochemical screening, and pharmacological properties. Furthermore, the bioactivities of isolated constituents and toxicological effects of *Brassica* seeds are also touched upon.

## 2. Traditional and Agronomic Uses of *Brassica* Seeds

*Brassica* seeds possess enormous bioactive compounds associated with a wide range of biological properties. Thanks to these virtues, *Brassica* plant seeds have been naturalized and adapted for use in agronomy and medicine [15,22]. Indeed, due to their high concentration of glucosinolates, seeds have been traditionally used as a spicy food condiment (Dijon mustard in France) [23,24]. Moreover, nonfood applications for seeds have become more popular, especially in the cosmetic and pharmaceutical industries. In traditional and modern medicine, mustard seeds are employed in various folk remedies as an appetizer, aperitif, digestive stimulant, laxative, expectorant, and antiseptic agent to treat gastrointestinal, respiratory, and skin diseases, as well as arthritis, foot aches, rheumatism, and lumbago [15,25]. In addition, because seeds are rich in proteins, they form a vital component of the food supply for pigs, poultry, and other types of livestock, in addition to aquaculture [26,27]. It has also been proven that *S. alba* seed meal is able to suppress weeds, while *B. juncea seed* meal is employed as a broad-spectrum pesticide against fungi, insects, and nematodes [28]. Moreover, seeds play a crucial role in the soil enrichment and fertilization process due to the favorable C/N ratio, the high nitrogen-rich protein content (20–45%), and the presence of essential nutrients including phosphorus (1%), potassium (1%), calcium (1%), magnesium (0.5%), sulfur (0.5 to 2%), zinc (100 mg/kg of total dry matter), manganese (100 mg/kg of total dry matter), and copper (10 mg/kg of total dry matter) [29]. Similarly, soil amendment with 10 D intermediate doses (30 t/ha) of *B. carinata* seed meal improved soil fertility by enriching total organic carbon, humified carbon, and phosphorus without adverse effects on microorganisms [30]. With 87% coagulation activity, an active coagulant napin protein isolated from *Brassica* seeds (*B. nigra*) was described as a helpful method to treat pond water turbidity [31].

## 3. Functional Ingredients of *Brassica* Genus Seeds

### 3.1. Edible Oil Profile

Vegetable oils rich in essential unsaturated fatty acids (UFAs) are an indispensable element of the human diet and are often suggested by nutritionists [32]. *Brassica* plants are well known for their seeds’ richness in edible oil, with an average content in *B. napus*, *B. juncea*, and *B. rapa* ranging from 45% to 50%. The oil content variability is attributable to genetic variances in *Brassica* species, environmental conditions, and agricultural practices [33]. Among all the *Brassica* species, *B. napus*, rapeseed, is one of the most important sources of edible oil. According to the United States Department of Agriculture (USDA report, January 2015), *B. napus* is the second largest produced oilseed crop worldwide with 71.94 million MT and the third largest source of vegetable oil. Indeed, the average oil content of *B. rapa* (47.30%) and *B. napus* (46.40%) was significantly higher compared to that of *B. carinata* (40%) and *B. nigra* (37.68%). Additionally, the general composition of oils from different *Brassica* seeds consists of seven major fatty acids: palmitic (C16:0), stearic (C18:0), oleic (C18:1), linoleic (C18:2), linolenic (C18:3), eicosanoic (C22:0), and erucic (C22:1) acids. *B. napus*, *B. rapa*, and *B. carinata* naturally accumulate high amounts of monounsaturated fatty acids, mainly erucic acid (C22:1ω9) (40–50%), while other species such as *Sinapis alba* and *B. nigra* have a moderate content of this fatty acid (Table 2).

This oil’s high concentration of erucic acid (about 40%) renders it unsuitable for human consumption [42]. Thus, the removal of erucic acid is required before rapeseed oil can be consumed by humans. The low-erucic-acid and low-glucosinolate cultivar is called “canola” in North America and “double-low” or “00” rapeseed in Europe. The oil extracted from double-low rapeseed is generally recognized as safe by the FDA [43]. It was demonstrated that mature oilseed rape seeds oil from double-low quality oilseed rape (low in erucic acid (up to 2% in consumption seeds) and low glucosinolate level (up to 25 μmol/g of seeds)) contained 60% monounsaturated oleic acid (C18:1), 30% polyunsaturated fatty acids (20% linoleic acid (C18:2) and 10% linolenic acid (C18:3)), 2% eicosenoic acid (C20:1), 7% saturated fatty acids (mainly palmitic (C16:0) and stearic acids (C18:0)), and 1% other acids [44]. As a result, double-low oil contained a high amount of oleic and linoleic acids, making it significantly healthier [45]. Oleic acid is suitable for low-cholesterol diets, for frozen food preparation, and to improve the stability of cooking and frying oils [46]. Moreover, linoleic (omega-6) acid reduces cholesterol and triglyceride levels and improves the viscosity of blood cells [33]. Linoleic acid (omega-6) and α-linolenic acid (omega-3) are essential fatty acids [47]. Furthermore, the low level of saturated fatty acids (SFAs) is beneficial for human health as their high consumption reduces the risk of cardiovascular diseases [48], while oils with a high polyunsaturated fatty acid (PUFA) content are more sensitive to lipid oxidation [49].

### 3.2. Proteins, Minerals, and Secondary Metabolites

Several research studies have been undertaken to assess the chemical composition of the seeds of *Brassica* genus species. In addition to their high oil content, *Brassica* seeds exhibited a high content of carbohydrates, fat, dietary fiber, and proteins, especially in *B. juncea*, *B. napus*, and *B. rapa* seeds [50,51] (Table 3). Indeed, there is a growing trend to isolate proteins from *Brassica* seeds, e.g., from canola seed meal to be used in human food [52] and as a coproduct of oil recovery [53]. An acyl carrier protein [54] and a ~60 kDa aminopeptidase enzyme, with maximum activity at pH 6.5 and temperature of 40 °C for Phe-pNA as a substrate, were characterized and purified from maturing seeds of *B. napus* [55]. Furthermore, from *B. napus* var. *oleifera* seeds, a new low-molar-mass trypsin serine proteinase iso-inhibitor, 5-oxoPro1-Gly62-RTI-III (6767.8 Da), was isolated [56]. Mustard seeds are very rich in protein of low value due to the presence of napin seed storage proteins, which are difficult to digest and have been identified as major allergens in humans [57,58]. In seed meal, four candidate napin genes in R-o-18 and 10 in Chiifu-401 were identified with high sequence similarity to *Arabidopsis thaliana* 2S albumin-like napin genes, which might be responsible for a high prevalence of food allergies [59]. From *B. rapa* seeds, 7S globulin-like *vicilin* SSPs were identified as the dominant seed storage proteins in the mature seed along with 2S albumin-like napin seed storage proteins (SSPs) and 11/12S globulin-like *cruciferin SSPs* [60]. In addition to napin seed storage proteins, three allergens were found in *S. alba*: Sin a2 (cruciferin), Sin a3 (non-specific lipid transfer protein), and Sin a4 (profilin) [61].

Chemical investigation of seeds has also revealed the presence of phytonutrients such as trace minerals like phosphorus [39], vitamin E [62], carotenoids, and tocopherols [63] (Table 3). 

Moreover, the aqueous and organic extracts of *Brassica* plant seeds have been found to contain considerable amounts of bioactive phytochemical compounds such as volatile oil, glycosides, reducing sugar, polyphenols, phenolic acids, flavonoids, alkaloids, saponins, terpenoids, tannins, and glucosinolates. The major chemical constituents in *Brassica* seeds as reported in the relevant literature are quantitatively mentioned in Table 3. It is clear that the specific molecules and their content in seeds vary greatly within species, varieties, and the extracting solvent. Thus, for example, as demonstrated in Table 3, alkaloids, saponins, and tannins are present only in *B. nigra* and *B. juncea* seeds [64,65,66]. In addition, studies have found that *Brassica* seeds are a valuable source of polyphenols. Vanillin, catechin, and quercetin have also been detected in *B. juncea* L. Czern seed extract with a high content of catechin, followed by vanillin [62].

Within the polyphenol richness, *Brassica* seeds are characterized by the presence of glucosinolates, especially in *B. campestris* [67] and *B. napus* [39], along with a high value in the seed meal of *B. rapa* L. (6.0 g/kg) [51]. The concentration of glucosinolates changes depending on the species, habitat, location, stage, and plant component [68,69,70]. High glucosinolate content gives vegetable oil its pungent and distinctive flavor and makes it less healthy [71]. *B. napus* seeds are dominated by aliphatic glucosinolates, representing between 91% and 94% in the different groups. The main aliphatic glucosinolates are progoitrin and gluconapin. Progoitrin ranges from 30.11 to 71.57 μmol/g dry weight (DW) in oilseed crops and root vegetable crops, respectively, whereas gluconapin, the second glucosinolate in abundance in the seeds, showed the highest content in forage crops with 30.17 μmol/g DW. Indole and aromatic glucosinolates are less abundant in seeds (less than 10% of the total glucosinolate content) [72]. Sinigrin was detected in *B. juncea* with a high content in 50% acetonitrile Dolsan mustard seeds extract with 53.77 mg/g [73].

**Table 3:** Chemical composition of *Brassica* spp. seeds, their derivatives (oil, meal, and cake), and sprouts obtained with different extracting solvents (%).

Chemical Composition	Species	Subspecies/var	Sample Analyzed	Extracting Solvent	Content	Reference
Volatile oil	*B. nigra*		Seeds	H_2_O	25.13% (*w*/*w*)	[64]
Fat	*B. juncea*		Seed meal	nf	2.8% (*w*/*w*)	[50]
	*B. napus*	Canola	Seed meal	nf	2.9% (*w*/*w*)	
Protein	*B. nigra*		Seeds	nf	24.70% (*w*/*w*)	[64]
	*B. oleracea*	*italica* cv. *Legacy*	Seeds	nf	27.29% (*w*/*w*)	[36]
	*B. rapa* L.	*Rapa Catozza Napoletana (RCN)*	Seed meal	nf	38.2% (*w*/*w*)	[51]
	*B. carinata*		Defatted cake	nf	24.6 to 35.4% (*w*/*w*)	[74]
	*B. juncea*	Canola	Seed meal	nf	47.4% (*w*/*w*)	[75]
			Seed meal	nf	41.7% (*w*/*w*)	[50]
	*B. napus*	Canola	Seed meal	nf	41.5% (*w*/*w*)	
			Seed meal	nf	48.6, 49.8% (*w*/*w*)	[39,75]
	*B. hirta*		Seeds	H_2_O	0.77% (*w*/*w*)	[76]
	*B. campestris*		Wild meal	nf	26% (*w*/*w*)	[67]
			Dehulled, defatted meal	nf	48% (*w*/*w*)	
Carbohydrates	*B. nigra*		Seeds	nf	35.40% (*w*/*w*)	[64]
	*B. oleracea*	*italica* cv. *Legacy*	Seeds	nf	58.89% (*w*/*w*)	[36]
Dietary fiber	*B. juncea*	*Canola*	Seed meal	nf	25.8% (*w*/*w*)	[75]
			Seed meal	nf	27.7% (*w*/*w*)	[50]
	*B. napus*	Canola	Seed meal	nf	33.8% (*w*/*w*)	
			Seed meal	nf	26.4%, 24.1% (*w*/*w*)	[75]
Crude fiber	*B. oleracea*	*italica* cv. *Legacy*	Seeds	nf	15.47% (*w*/*w*)	[36]
	*B. nigra*		Seeds	nf	0.30% (*w*/*w*)	[64]
	*B. campestris*		Wild meal	nf	13.4% (*w*/*w*)	[67]
			Dehulled and defatted meal	nf	3.8% (*w*/*w*)	
Oligosaccharides	*B. napus*		Seed meal	nf	2.1% (*w*/*w*)	[39]
Glycosides	*B. nigra*		Seeds	H_2_O	20.01% (*w*/*w*)	[64]
Reducing sugar	*B. nigra*		Seeds	H_2_O	5.56% (*w*/*w*)	[64]
Starch	*B. juncea*		Seed meal	nf	3.4% (*w*/*w*)	[50]
	*B. napus*	Canola	Seed meal	nf	1% (*w*/*w*)	
	*B. napus*		Seed meal	nf	2.3% (*w*/*w*)	[39]
Nonstarch polysaccharides	*B. napus*		Seed meal	nf	17.5% (*w*/*w*)	
Sucrose	*B. juncea*	Canola	Seed meal	nf	9.2% (*w*/*w*)	[75]
	*B. juncea*		Seed meal	nf	6.9% (*w*/*w*)	[50]
	*B. napus*	*canola*	Seed meal	nf	5.6% (*w*/*w*)	
	*B. napus*		Seed meal	nf	7.5%, 10.2% (*w*/*w*)	[39,75]
Moisture	*B. nigra*		Seeds	nf	4.16% (*w*/*w*)	[64]
	*B. campestris*		Wild meal	nf	4.8% (*w*/*w*)	[67]
Ash	*B. nigra*		Seeds	nf	5.14% (*w*/*w*)	[64]
	*B. oleracea*	*italica* cv. *Legacy*	Seeds	nf	4.45% (*w*/*w*)	[36]
	*B. campestris*		Wild meal	nf	4.4% (*w*/*w*)	[67]
			Dehulled and defatted meal	nf	7% (*w*/*w*)	
Phosphorus	*B. napus*		Seed meal	nf	1.14% (*w*/*w*)	[39]
Non-phytate phosphorus	*B. napus*		Seed meal	nf	0.83% (*w*/*w*)	
Vitamin E	*B. juncea* L.	*Czern*	Seeds	80% methanol, 20% H_2_O	0.08% (*w*/*w*)	[62]
Vitamin C	*B. oleracea*	*italica* Green King variety	Seeds	70% methanol	0.27% AAE (*w*/*w*)	[77]
α-tocopherol	*B. nigra*		Seeds	Hexane, ethyl acetate, and methanol	0.11% (*w*/*v*)	[63]
	*B. oleracea* L.	var. *acephala*	Oil	nf	70% (*w*/*w*)	[35]
Total phenolic	*B. nigra*		Cold-press oil	nf	0.01% GAE (*w*/*v*)	[78]
	*B. oleracea*	*italica cv. Legacy*	Seeds	Methanol/water (80:20)	0.89% GAE (*w*/*w*)	[36]
		*italica* Green King variety	Seeds	70% methanol	1.66% GAE (*w*/*w*)	[77]
			Seeds	Methanol	0.39–0.46% GAE (*w*/*w*)	[79]
	*B.rapa* L.		RCN seed meal	nf	1.30% (*w*/*w*)	[51]
	*B. tournefortii*	*Gouan*	Oil	nf	1.61% GAE (*w*/*w*)	[80]
Total phenolic	*B. carinata*		Defatted cake	Methanol	0.04–0.13% (*w*/*w*)	[74]
	*B. hirta*		Seeds	H_2_O	0.63% (*w*/*w*)	[76]
			Cold-press oil	nf	0.02% GAE (*w*/*v*)	[78]
	*B. juncea*	*Czern*	Seeds	70% ethanol	2.77% TAE (*w*/*w*)	[81]
			Seeds	nf	0.12% GAE (*w*/*v*)	[65]
			Dolsan mustard seeds (DMS)	50% acetonitrile (ACN)	40.43% GAE (*w*/*w*)	[73]
	*B. napus*	Canola	Seeds	80% methanol	46.23% (*w*/*w*)	[82]
		Canola	Defatted oilseed cakes	Methanol/acetone/water (MAW)	2.11% GAE (*w*/*w*)	[83]
Total flavonoid	*B. nigra*		Seeds	H_2_O	6.57% (*w*/*w*)	[64]
			Cold-press oil	nf	0.002% CE (*w*/*v*)	[78]
	*B. oleracea var.*	*italica* Green King variety	Seeds	70% methanol	0.37% CE (*w*/*w*)	[77]
		*italica cv. Legacy*	Seeds	Methanol/water (80:20)	3.3% QE (*w*/*w*)	[36]
			Seeds	Methanol	0.26–0.40% RE (*w*/*w*)	[79]
	*B. juncea*		DMS	50% ACN	39.53% QE (*w*/*w*)	[73]
			Seeds	nf	2.48% RE (*w*/*w*)	[65]
		*Czern*	Seeds	70% ethanol	12.68% QE (*w*/*w*)	[81]
	*B. napus*	Canola	Seeds	80% methanol	7.54% (*w*/*w*)	[82]
		Canola	Defatted oilseed cakes	MAW	0.04% LUE (*w*/*w*)	[83]
	*B. hirta*		Cold-press oil	nf	0.001% CE (*w*/*v*)	[78]
Vanillin	*B. juncea*	*Czem*	Seeds	80% methanol, 20% H_2_O	0.21% (*w*/*w*)	[62]
Catechin					0.42% (*w*/*w*)	
Quercetin					0.01% (*w*/*w*)	
Alkaloids	*B. nigra*		Seeds	H_2_O	20.58% (*w*/*w*)	[64]
	*B. juncea*		Seeds	10% acetic in ethanol	2.25% (*w*/*w*)	[65]
Tannins	*B. nigra*		Seeds	H_2_O	15.05% (*w*/*w*)	[64]
	*B. juncea*		Seeds	nf	7.75% TAE (*w*/*v*)	[65]
Saponins	*B. nigra*		Seeds	H_2_O	12.82% (*w*/*w*)	[64]
	*B. juncea*		Seeds	80% methanol	4.25% Diosgenin equivalent (*w*/*w*)	[65]
Terpenoids	*B. juncea*		Seeds	Ethanol	5.40% (*w*/*w*)	
Carotenoids	*B. nigra*		Seeds	Hexane, ethyl acetate, and methanol	1.51% (*w*/*v*)	[63]
Glucosinolates	*B. oleracea*	*italica*	Sprouts	nf	0.40% (*w*/*w*)	[84]
		*italica*	Seeds	Aqueous methanol (80% *v*/*v*)	1.01% sinigrin equivalent (*w*/*w*) to 2.09% sinigrin equivalent (*w*/*w*)	[85]
	*B. napus*		Seed meal	nf	20.8% (*w*/*w*)	[39]
			Seeds	Methanol, lead acetate 0,3 M, water	Oilseed group: 2.3% (*w*/*w*) Forage group: 4.88% (*w*/*w*)	[72]
	*B. rapa* L.		RCN seed meal	nf	0.6% (*w*/*w*)	[51]
	*B. campestris*		Dehulled and defatted meal	nf	2.3% (*w*/*w*)	[67]
Sinigrin	*B. juncea* L.	*Czern*	Seeds	80% methanol, 20% H_2_O	0.08% (*w*/*w*)	[62]
	*B. juncea*		DMS	50% ACN	5.38% (*w*/*w*)	[73]

nf: not found in original publication, AAE: ascorbic acid equivalent, GAE: gallic acid equivalent, TAE: tannic acid equivalent, CE: catechin equivalent, QE: quercetin equivalent, LUE: luteolin equivalent.

### 3.3. Aqueous, Organic Extracts and Essential Oil Phytochemical Profile

Due to the presence of major phytochemicals compounds in the seeds of *Brassica* genus, many studies have been oriented to their identification and quantification using different methods including high-performance liquid chromatography (HPLC) coupled to DAD or UV detectors and HPLC fluorescence, reverse-phase HPLC (reverse-phase high-performance liquid chromatography), gas chromatography, qualitative LC–ESI-MS^n^ analysis, gas chromatography coupled to mass spectrometry (GC–MS), GC-FID, NMR spectroscopy, ^13^C-NMR, ^1^H-NMR, high-resolution electrospray ionization mass spectrometry (HR-ESI), LH-20 chromatography, and Sephadex LH-20. The extraction solvents used are typically hexane, dichloromethane, petroleum ether, methanol, ethyl alcohol, ethyl acetate, acetone, water, and trifluoroacetic acid (TFA).

The constituents identified and quantified from different *Brassica* plant seeds are represented and detailed in Table 4. 

Phenolic compounds, made up of aromatic rings with hydroxyl groups, represent the most abundant secondary metabolites in plant with more than 8000 identified structures. Plants generate phenolics via the shikimic pathway [86]. As reported in Table 4, different polyphenols including phenolics, flavonoids (flavonols, flavones, flavan-3-ols, anthocyanidins, flavanones, isoflavones, and others), and non-flavonoids (phenolic acids, hydroxycinnamates, stilbenes, and others) have been identified and quantified in *B. nigra*, *B. oleracea* var. *acephala*, *B. oleracea* var. *costata*, *B. rapa* L., *B. napus*, and *B. alba* seeds. The most abundant and diversified groups of polyphenols in *Brassica* species are flavonoids (mostly flavonols and anthocyanins) and hydroxycinnamic acids [86]. Flavonols are the major representative of flavonoids. *Brassica* crops’ principal flavonols, quercetin, kaempferol, and isorhamnetin, are most typically found as O-glycosides [87]. In *Brassica* vegetables, the most common hydroxycinnamic acids are p-coumaric (4-hydroxycinnamic), sinapic (3,5-dimethoxy-4-hydroxycinnamic), and ferulic acids (4-hydroxy-3-methoxycinnamic) [11,88]. In *Brassica* seeds, the most prominent phenolic compounds were determined to be sinapic acid derivatives such as 1-O-β-D-glucopyranosyl sinapate and 1,2-di-O-sinapoyl-β-D-glucose [82].

The seed composition profile of *Brassica* differs with the variety, extraction solvent, and detection or quantification methods used. Compared to other *Brassica* species, *B. napus* seeds have been shown to be particularly rich in polyphenols through the detection of 91 flavonoids and hydroxycinnamic acid derivatives and the identification of 78 compounds, of which 55 were first reported in *B. napus* L. var. *napus* and 24 were first detected in the genus *Brassica* [89]. In another phytochemical investigation in *B. napus* seeds from different winter type oilseed rape genotypes (Aviso (00), CMB1039 (00), Doublol (00), JetNeuf (0þ), and PR3984 (00)), 13 different flavonoids were identified by liquid chromatography coupled to electrospray ionization mass spectrometry (LC–ESI-MS^n^) and characterized for the first time in the seed coat of *B. napus*, and isorhamnetin-hexoside-sulfate and isorhamnetin-sinapoyltrihexoside were newly identified in *Brassica* spp. [90]. 

Glucosinolates are the major *Brassica* seed phytochemicals. Aliphatic, indolic, and aromatic glucosinolates are the three principal chemical groups of GLSs, classified according to the amino-acid precursor, forming more than 130 types. Aliphatic GLSs are mainly derived from methionine but also from alanine, leucine, isoleucine, or valine, while the indolic GLSs are tryptophan-derived compounds and aromatic GLSs are phenylalanine- and tyrosine-derived compounds [91]. Detecting and identifying GLS directly or indirectly mainly depend on the existence (intact) or absence (desulfo) of the sulfate group. GLSs are hydrolyzed by myrosinase after cell disruption and by the gut microbiota [92,93]. Isothiocyanates (ITCs), nitriles (CNs), epithionitriles (EPNs), and thiocyanates are the common GLS breakdown products [69,94]. Myrosinase is inactivated at 60 °C and 700 MPa [95]. GLS content and profile vary with *Brassica* species and organ, with a high amount in the reproductive system including florets, flowers, and seeds. Variations in the profile of glucosinolates and their hydrolysis products of different seeds have been detected, as shown in Table 4. Thus, for example, it has been reported that the major glucosinolates found in *B. rapa* L. seeds were progoitrin, glucoraphanin, gluconapin, and 4-hydroxyglucobrassicin [51], whereas *B. oleracea italica*, the ethyl acetate oil of *B. juncea raya*, and *B. campestris* were characterized by the presence of glucosinolates hydrolytic products such as allyl isothiocyanate, 2-phethyl isothiocyanate, 3-butenyl isothiocyanate, and 3-(methylthio) propyl isothiocyanate [96,97]. Glucoerucin and glucoraphanin were the two major glucosinolates found in broccoli seeds [85]. Moreover, the indole glucosinolate, 4-hydroxy-3-indolylmethyl glucosinolate, was purified and identified as a major constituent of cabbage seed and rapeseed [98]. In addition, it was found that sulforaphane is an abundant isothiocyanate of broccoli, containing 49.77 mg/g [96]. Given the richness of the seeds in sulforaphane (SFN), research work has focused on its isolation and purification in *Brassica oleracea* L. var. *rubra* seeds [99] and *B. oleracea* L. var. *italica* (broccoli) seeds, which were purified to 186 mg of sulforaphane from 850 mg of the ethyl acetate seed meal extract by solid-phase extraction, preparative high-performance liquid chromatography, and high-speed countercurrent chromatography (HSCCC) before being characterized by MS and H- or C-NMR [100,101].

A phytochemical investigation conducted on *B. napus* seeds led to the isolation by NMR spectroscopy of two new nitrogenous compounds, (2S)-2-sinapoyl-4-pentenenitrile and brassicalkaloid A, together with four known alkaloids, coixspirolactam C, 1H-indole-3-acetonitrile, 3-indolealdehyde, and indole-3-acetonitrile-2S β-D-glucopyranoside [102].

The phytochemical screening of *B. oleracea* L. var. *acephala* seeds demonstrated the presence of 13 carotenoids, among which all-elutein was the main component [35].

Furthermore, an investigation conducted on broccoli seeds (*B. oleracea* L. var. *italica* cv. *Legacy*) revealed a large number of amino acids with a predominance of glutamic acid (72.83 mg/g fresh weight (FW)), asparagine (51.81 mg/g FW), serine + histidine (34.16 mg/g FW), and proline (23.29 mg/g FW) [36].

Additionally, in *B. oleracea L.* var. *costata DC* seeds, seven organic acids were identified and quantified by HPLC-UV with a maximum concentration of ascorbic (8546 mg/kg, dry basis (DB)) followed by citric (4685 mg/kg, DB), and malic + quinic (3049 mg/kg, DB) acids [103].

Moreover, in the same analyzed sample, a variable profile was demonstrated in the metabolite families. Indeed, the GC–MS analysis of *Brassica napus* petroleum ether extract revealed the presence of different chemical compounds such as acetic acid butyl ester, docosane 11-decyl, 2-pentanone 4-hydroxy-4 methyl, bicyclooct-2-ene-4α, 6α-carbolactone, benzene 1,4-dimethyl, 2-methyl-5-phenyl-5-pentanonenitrile, 5,8,11,14-eicosatetraenoic acid methyl ester, 3,4-dihydrothienyl-5-carboxtthiol, 1-butene 4-isothiocyanato, 7,7-dimethyl-tetracycloheptane, 9-methyl-l0-tetradecen-1-ol acetate, and 9-hydroxy-1-methyl-1,2,3,4-tetrahydro-8H-pyrido (1,2α) pyrazin-8-one [104].

Moreover, the essential oil composition of *Brassica* seeds is represented in Table 4. The essential oil of *B. napus* seeds was dominated by 2-phenyl ethyl isothiocyanate (39.2%) followed by bicyclohept-6-en-1-yl-tert-butyl ether (13.7%), 2-(allylthio) 1-nitrobutane (12.8%), 4-bromo-3-phenylbut-2-enoate (9.8%), 1,3,6,10-cyclotetradecatetraene 3,7,11-trimethyl-l4-(1-methylethyl) (5.2%), 1-butene 4-isothiocyanato (4.8%), cyclohexane 1,12-[1-(2, 2-dimethylbutyl)-1,3-propanediyl] bis (1.2%), and 4 trifluroacetoxytetradecane (1.1%) [104]. In research conducted to evaluate the *Sinapis alba* seeds, 14 components in the essential oil from mustard seeds representing 97.94% of the total amount were identified. The predominant component of the essential oil was allyl isothiocyanate (AITC), representing 71.06% [105]. The GC–MS analysis revealed that the main components in the essential oil of *B. nigra* seeds were di-(9-octadecenoyl)-glycerol (42.16%), 9,12-octadecadienoyl chloride, (Z,Z)-(41.40%), and hexadecanoic acid, 1-(hydroxymethyl)-1,2-ethanediyl ester (13.20%), while the main components in the essential oil of *B. hirta* were cyclopropanenonanoic acid (48.70%), 2-[(2-butylcyclopropyl) methyl]-methyl ester, and hexadecanoic acid, 1-(hydroxymethyl)-1,2-ethanediyl ester (42.08%) [78].

**Figure 1:**
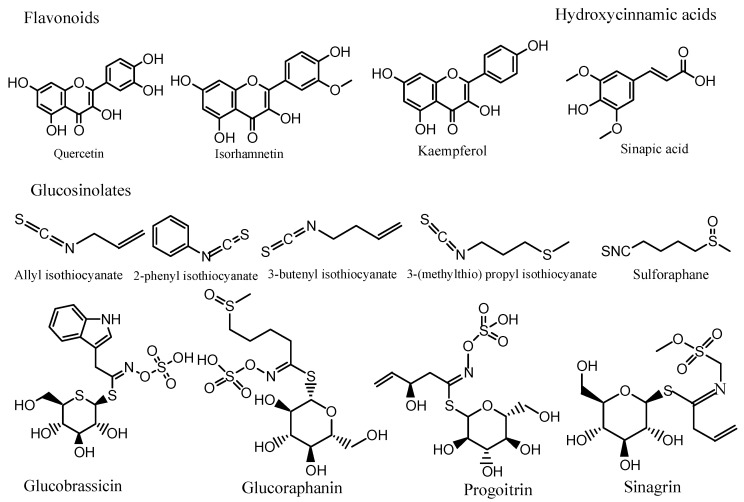
Molecular structures of the main bioactive compounds present in *Brassica* spp. seeds.

Overall, several phytochemistry studies have investigated the phytochemical compositional diversity in *Brassica* spp. seeds, and a wide range of bioactive compounds (polyphenols, glucosinolates, carotenoids, alkaloids, amino acids, fatty acids, and organic acids) have been identified and quantified using different analytical approaches such as HPLC-UV, HPLC-DAD, LC–MS, LC–MS/ESI, GC, and GC–MS. Polyphenols such as hydroxycinnamic acids and flavonoids were the most common. Flavonoids (flavonols) were mostly found as quercetin, kaempferol, and isorhamnetin derivatives. Glucosinolates were mostly found in aliphatic forms. The hydrolytic glucosinolates products with the highest content were allyl isothiocyanate, 2-phethyl isothiocyanate, 3-butenyl isothiocyanate, and 3-(methylthio) propyl isothiocyanate. Sulforaphane, a powerful anticancer isothiocyanate, was also identified and quantified in *Brassica* seeds (Figure 1).

However, available data of phytochemical screening of diverse *Brassica* spp. seeds are still limited, and different understudied phytochemicals require more detailed characterization.

The biosynthesis, variability, and abundance of *Brassica* seed phytochemicals depend on genotype, environmental factors, germination, and degree of maturity [106]. Indeed, germination improved the phenolic compounds content by 49% and 44% in *Sinapis alba* and *Brassica nigra* seeds, respectively, as well as in broccoli seeds [36,107]. Furthermore, asparagine, proline, and glutamic acid were much more abundant in broccoli seeds after germination [36]. Consequently, germination is a simple and effective approach to boost seed nutrients. Yet, the germination time reduced the broccoli seed total flavonoid content, probably resulting from the moisture accumulation [79].

As shown in Table 3 and Table 4, the phytochemical profile variation in different *Brassica* seed species is also dependent on the extraction solvent, the extraction methods, and the detection and quantification approaches. Sample preparation and extraction methods are crucial steps. Acetone, methanol, ethanol, ethyl acetate, and their mixtures with water are the most employed solvents in plant phenolic extraction [11]. Indeed, equal parts water and acetone has a better effect on the phenolic compounds extraction of mustard seed [108]. Generally, the extraction of phenolic compounds is most effective using solvents with high polarity [109]. Numerous conventional and advanced extraction techniques such as maceration, soxhlet, microwave-assisted extraction (MAE), and ultrasound-assisted extraction (UAE) are applied for the phytochemical screening of *Brassica* seeds. It was reported that hydrodistillation increased the canola oil extraction level, while, in Indian mustard seed, soxhlet extraction with petroleum ether raised the oil content to 37% with strong antioxidant potential [104,110]. Furthermore, it was reported that the inactivation of myrosinase by a heating process (30 min at 60 °C), followed by methanol/water extraction and solid-phase extraction (SPE), is the preferable strategy for obtaining high GLS yield. The myrosinase inactivation by a heating process and high methanol concentration prevented the GLS enzymatic hydrolysis [111].

The most common analytical techniques applied in *Brassica* seed phytochemical profile analysis are GC and HPLC. A variable profile has been demonstrated within the *Brassica* spp. GC and GC–MS are applied to identify the volatile organics directly or indirectly after derivatization. They have been used to characterize the GLS breakdown products and fatty acids [78,97]. However, some isothiocyanates, such as sulforaphane, are thermally unstable and, consequently, GC is inadequate for their evaluation [36,112]. Additionally, due to the non-volatility or thermal instability of some GLSs, GC is not appropriate for their direct analysis and, consequently, must be converted into volatile derivatives which may be not suitable with some GLS structures [111]. HPLC is, thus, the preferred method with a desulfation step to reduce the polarity for the separation by reverse-phase chromatography (RPC) [113]. Therefore, as shown in Table 4, HPLC is the frequently used technique for GLS and amino-acid analysis in *Brassica* seeds [36,114]. However, the lack of standards of some compounds is a limiting factor of HPLC viability. Thus, to improve phytochemical detection and quantification, the combination of HPLC with the highly sensitive method MS (LC–MS) is needed as a rapid, selective and sensitive approach [35,115]. Yet, due to its insufficient precision and accuracy to identify some GLS peaks (e.g., similar *m*/*z* of both glucoiberin (*m*/*z* 422.0255) and gluconasturtiin (*m*/*z* 422.0585)) and the lack of reference materials, LC–MS use remains limited [111]. LC–tandem mass spectrometry (LC–MS/MS) can resolve this issue since fragmentation improves both selectivity and sensitivity [90,103]. Recently, ultra-HPLC–MS/MS (UHPLC–MS/MS) data were used to create a new platform (GLS-Finder) useful for intact GLS identification in 49 popular *Brassica* vegetables [116]. However, certain reviewers stated that the number of GLS/isomer chromatographic peaks was higher than the known GLS number of every extract, which should be verified with the published compositions [91,117].

Moreover, NMR has been utilized as the most effective technique for the final confirmation of the identified and isolated phytochemical compound structure [101,102,118].

The validation of the extraction methods and the analytical techniques is crucial for the precision and the accuracy of the phytochemical profile data.

**Table 4:** Bioactive compounds identified from *Brassica* seed/derivative extracts and essential oil using different analytical techniques (%).

Chemical Composition	Species	Subspecies/var	Sample Analyzed	Extracting Solvent	Separation and Detection Methods	Bioactive Compounds	Reference
Polyphenols	*B. nigra*		Seeds		UHPLC–MS/MS	4-hydroxybenzoic acid, caffeic acid, p-coumaric acid, ferulic acid, sinapic acid, and procatechuic acid	[119]
	*B. oleracea*	var. *acephala*	Oil		GC-FID, MS, HPLC-DAD, HPLC–MS, HPLC fluorescence	11 polyphenols, 5 flavonoids, and 6 hydroxycinnamic acids	[35]
		var. *costata*	Seeds	H_2_O	Reverse-phase HPLC-DAD–MS/MS-ESI and HPLC-DAD	13 phenolic compounds: 2 sinapoylgentiobiose isomers (sinapoylgentiobiose (0.03% (*w*/*w*)) and sinapoylgentiobiose isomer (0.03% (*w*/*w*))), 3 sinapoylglucose isomers (1-sinapoylglucose isomer (0.04% (*w*/*w*)), 1-sinapoylglucose isomer (0.04% (*w*/*w*)), and 1-sinapoylglucose (0.07% (*w*/*w*))), kaempferol-3 (sinapoyl) sophorotrioside-7-glucoside, sinapoylcholine, kaempferol-3,7-diglucoside-4’-(sinapoyl) glucoside, 3 disinapoylgentiobiose isomers (1,2-disinapoylgentiobiose isomer (0.02% (*w*/*w*)), 1,2-disinapoylgentiobiose isomer (0.04% (*w*/*w*)), and 1,2-disinapoylgentiobiose (0.10% (*w*/*w*))), 1,2,2’-trisinapoylgentiobiose, and 1,2-disinapoylglucose	[103]
	*B. rapa* L.	*RCN*	Seed meal	70% methanol	LC–MS	Polyphenols: flavonol and hydroxycinnamic derivatives: K−3−O-(methoxycaffeoyl) sophotrioside−7−O-glc, K−3−O-sophotrioside−7−O-glc, Q−3−O-(coumaroyl) sophoroside −7−O-glc, K−3−O-(sinapoyl) sophotrioside−7−O-glc, I−3−O-(cumaroyl) sophotrioside−7−O-sophoroside, I-3,7−O-di-glc, I−3−O-glc−7−O-sophoroside, caffeoyl derivative, K−3−O-sophoroside, Q−3−O-sophoroside, K−3−O-(feruloyl) sophoroside, Q−3−O-glc, 1,2-disinapoylgentiobioside, 1-sinapoyl-2-feruloylgentiobioside, K−3−O-glc, and I−3−O-glc	[51]
	*B. juncea*		Seeds		UHPLC–MS/MS	4-hydroxybenzoic acid, syringic acid, p-coumaric acid, ferulic acid, sinapic acid (0.02% (*w*/*w*), procatechuic acid, and kaempferol	[119]
				30% etthanol	LC–MS/MS	Caffeic acid, p-coumaric acid, epigallocatechin gallate, myricetin, apigenin, quercetin-3-O-(caffeoyl)-glucoside, and quercetin	[120]
	*B. napus*	*spring oilseed rape (Napus* cv. *Drakkar)*	Seeds	Hexane–80% aq. methanol	Combination of high-field NMR spectroscopy and high-resolution electrospray ionization mass spectrometry (HR-ESI)	15 constituents: glucose, kaempferol glycoside esters, gentiobiose,, sinapine (sinapoylcholine), and sinapoylmalate; 1 of the glucose esters (1,6-di-O-sinapoylglucose), 2 kaempferol conjugates [4’-(6-O-sinapoylglucoside)-3,7-di-O-glucoside and 3-O-sophoroside-7-O-(2-O-sinapoylglucoside)], and 2 gentiobiose esters (1-O-caffeoylgentiobiose, and 1,2,6’-tri-O-sinapoylgentiobiose)are new plant products	[121]
		*winter type: Aviso (00), CMB1039 (00), Doublol (00), JetNeuf (0þ) and PR3984 (00)*	Seed coat	Methanol/acetone/water/TFA mixture (40:32:28:0.05 *v*/*v*/*v*/*v*)	LC–ESI-MS^n^	13 different flavonoids: (−)-epicatechin, 7 flavonols (quercetin-3-O-glucoside, quercetin-dihexoside, kaempferol-sinapoyl-trihexoside, isorhamnetin-3-O-glucoside, isorhamnetin-dihexoside, isorhamnetin-hexoside-sulfate, and isorhamnetin-sinapoyl-trihexoside), and 5 procyanidins	[90]
		*Canola*	Seeds	80% methanol	Sephadex LH-20 chromatography	Free phenolic compound: trans-sinapic acid (19.34% (*w*/*w*)); sinapic acid derivatives: 1-O-β-D-glucopyranosyl sinapate and 1,2-di-O- sinapoyl-β-D-glucose	[82]
		*cv.Yang 6*	Seeds	80% methanol	HPLC-PDA–ESI(−)-MS^n^/HRMS	Detection of 91 flavonoids and hydroxycinnamic acid derivatives: 6 flavanols and their oligomers, 39 kaempferol derivatives, 5 quercetin derivatives, 11 isorhamnetin derivatives, and 30 hydroxycinnamic acid derivatives	[89]
	*B. alba*		Seeds		UHPLC–MS/MS	4-hydroxybenzoic acid, apigenin, p-coumaric acid, ferulic acid, and sinapic acid (0.12% (*w*/*w*))	[119]
Glucosinolates	*B. oleracea*	*italica (broccoli)*	Seed isothiocyanates (ITCs)	Ethyl acetate	GC–MS	3-BITC (3-butenyl isothiocyanate) (13.85% (*w*/*w*)) and sulforaphane (4.98% (*w*/*w*))	[96]
		*Alboglabra (Chinese kale)*				3-BITC (7.76% (*w*/*w*))	
		*italica (broccoli)*	Seeds	H_2_O	HPLC	Aliphatic glucosinolates: sinigrin (0–1.94% (*w*/*w*)), progoitrin (0–5.51% (*w*/*w*)), glucoraphanin (0.12–6.18% (*w*/*w*)), gluconapin (0–0.7% (*w*/*w*)), glucoerucin, glucoiberin, and glucoiberverin. Indolic glucosinolates: glucobrassicin (0–0.17% (*w*/*w*)), and 4-hydroxy-glucobrassicin (0–0.33% (*w*/*w*))	[122]
		*italica* cv. *Legacy*	Seeds	Dichloromethane (DCM)	HPLC	Sulforaphane (0.36% (*w*/*w*))	[36]
	*B. rapa* L.	*Rapa L*	Seeds	DCM	GC–MS	Phenylethylbrassinin and 3-phenylpropionitrile	[123]
		*RCN*	Seed meal	70% methanol	LC–MS	(R)-2-Hydroxy-3-butenyl (progoitrin), 4-methylsulfinylbutyl (glucoraphanin), 3-butenyl (gluconapin), 4-hydroxy-3-indolylmethyl (4-hydroxyglucobrassicin), 4-pentenyl (glucobrassicanapin), 3-indolylmethyl (glucobrassicin), and N-methoxy-3-indolylmethy (neoglucobrassicin)	[51]
	*B. juncea*		Seeds	5% ethyl alcohol		Allyl isothiocyanate (0.48% (*w*/*w*))	[41]
		*raya*	Oil	Ethyl acetate	GC/GC–MS	GSLs hydrolytic products in ethyl acetate oil: allyl isothiocyanate (23% (*w*/*w*)), 2-phethyl isothiocyanate (~20% (*w*/*w*)), 3-butenyl isothiocyanate (18% (*w*/*w*)), 3-(methylthio) propyl isothiocyanate, allyl thiocyanate, and 1-isothiocyanato-3-methyl butane	[97]
				DCM	GC/GC–MS	GSLs hydrolytic products: phenethyl isothiocyanate (15.15% (*w*/*w*)), 4-pentenyl isothiocyanate (12.548% (*w*/*w*)), sec-butyl isothiocyanate, allyl isothiocyanate, and isothiocyanic acid	
		*Coss and Czern*	Seeds	Methanol	^13^C-NMR, ^1^H-NMR	3 native glucosinolates isolated: p-hydroxybenzyl glucosinolates, newly described in *B. juncea* seeds, and 2 new compounds 9-(methyl-sulfonyl) nonyl and 8-(methylsulfonyl) octyl glucosinolates	[118]
			Seeds	Double-distilled water (ddH_2_O)	HPLC	4 aliphatic GLSs: sinigrin, progoitrin, gluconapin, and glucoiberin, 4 indolic GLSs: glucobrassicin, neoglucobrassicin, 4-methoxyglucobrassicin and 4-hydroxy glucobrassicin, and one aromatic GLS: gluconasturtiin; sinigrin is the predominant GLS with 90% of total GLSs, followed by gluconapin	[114]
			Seeds	DCM	GC	Glucosinolates breakdown products (GBPs): 5 ITCs: [2-propenyl isothiocyanate, 3-butenyl isothiocyanate, 5-vinyl-1,3-oxazolidine-2-thione, 3-methyl sulfinylpropyl isothiocyanate, and 2-phenylethyl isothiocyanate], 2 CNs: [3-butenenitrile and 4-pentenenitrile], and 3 EPNs: [3,4-epithiobutanenitrile, 4,5-epithiopentanenitrile, and 3-hydroxy-4,5-epithiopentanenitrile]; 2-propenyl isothiocyanate is the predominant individual GBP with 51–98% of total GBPs, followed by 3-butenenitrile, 3,4-epithiobutanenitrile, 3,4-epithiobutanenitrile, and 3-butenyl isothiocyanate.	
	*B. hirta*	*Sinapis alba*	Seeds	5% ethyl alcohol		Allyl isothiocyanate (0.15% (*w*/*w*))	[41]
	*B. campestris*		Isothiocyanates (ITCs)	Ethyl acetate	GC–MS	3-BITC (3-butenyl isothiocyanate) contained (7.76% (*w*/*w*))	[96]
Carotenoids	*B. oleracea*	*var. acephala*	Seed oil		GC-FID, MS, HPLC-DAD, HPLC–MS, HPLC fluorescence	13 carotenoids, with all-elutein as the main component	[35]
Amino acids	*B. oleracea*	*italica cv. Legacy*	Seeds	Hydrochloric acid	HPLC	Glutamic acid (7.28% (*w*/*w*)), asparagine (5.18% (*w*/*w*)), serine + histidine (3.42% (*w*/*w*)), proline (2.33% (*w*/*w*)), threonine (1.88% (*w*/*w*)), leucine (1.37% (*w*/*w*)), valine (1.03% (*w*/*w*)), tyrosine (0.95% (*w*/*w*)), phenylalanine (0.87% (*w*/*w*)), isoleucine (0.8% (*w*/*w*)), glycine (0.74% (*w*/*w*)), methionine (0.42% (*w*/*w*)), arginine (0.39% (*w*/*w*)), and alanine (0.23% (*w*/*w*))	[36]
Fatty acids	*B. nigra*		Cold-press oil		GC–MS	Methyl erucate (38.23% (*w*/*w*)), 8,11,14-docosatrienoic acid, methyl ester (23.72% (*w*/*w*)), 11-eicosenoic acid, methyl ester (15.82% (*w*/*w*)), and methyl linoleate (10.13% (*w*/*w*))	[78]
	*B. hirta*		Cold-press oil		GC–MS	Methyl linoleate (68.19% (*w*/*w*)), methyl oleate (15.79% (*w*/*w*)), and hexadecanoic acid, methyl ester (10.51% (*w*/*w*))	
	*B. juncea*	*raya*	Oil	Ethyl acetate and DCM	GC/GC–MS	Octadecenoic acid (5.67–23.3% (*w*/*w*)), hexaicenoic acid (4.98–20% (*w*/*w*)), butanedioic acid (1.6–16% (*w*/*w*)), and nonanedioic acid (4.73% (*w*/*w*))	[97]
Organic acids	*B. oleracea*	*var. costata*	Seeds	H_2_O	HPLC-UV	7 organic acids: aconitic (0.02% (*w*/*w*)), citric (0.47% (*w*/*w*)), ascorbic (0.86% (*w*/*w*)), malic + quinic (0.31% (*w*/*w*)), shikimic, and fumaric acids	[103]
Sterols	*B. juncea*		Seeds		HPLC, GC, ^1^H-NMR	Sterol, 22-dehydrocampesterol [(24S)24-methylcholesta-5, E-22-dien-3β-ol], newly discovered in higher plants	[124]
Essential oil	*B. hirta*	*Sinapis alba*	Essential oil	H_2_O	GC–MS and GC-FID	2-methylbutyronitrile, 3-pentenenitrile, hexanal, furfural, 2-furanmethanol, cyclopropyl isothiocyanate, allyl isothiocyanate, isobutyl isothiocyanate, 3-butenyl isothiocyanate, benzene acetaldehyde, 3-methylbutyl isothiocyanate, 3-(methylthio) propyl cyanide, 2-phenylethyl cyanide, and 2-phenethyl isothiocyanate	[105]
			Cold-press oil		GC–MS	Cyclopropanenonanoic acid, 2-[(2-butylcyclopropyl) methyl] -, methyl ester (48.7% (*w*/*w*)), and hexadecanoic acid, 1-(hydroxymethyl)-1,2-ethanediyl ester (42. 08% (*w*/*w*))	[78]
	*B. oleracea*	var. *botrytis* (L.), *Romanesco group*	Seed/volatile oil	DCM	GC and GC–MS	Natalino variety: 43 compounds (99.7% (*w*/*w*)), contained 10 cyanides (88.1% (*w*/*w*)), 10 isothiocyanates (8.8% (*w*/*w*)), and 8 aldehydes (1.3% (*w*/*w*)) Campid oglio variety: 41 compounds (99.6% (*w*/*w*)) contained 10 cyanides (92.3% (*w*/*w*)), 10 isothiocyanates (6.2% (*w*/*w*)), and 6 aldehydes (0.4% (*w*/*w*)) Navona variety: 32 compounds (99.5% (*w*/*w*)) contained 9 cyanides (91.0% (*w*/*w*)), 6 isothiocyanates (7.5% (*w*/*w*)), and 6 aldehydes (0.6% (*w*/*w*)) Cyanides: 2-methylpropyl cyanide, but-3-enyl cyanide, 3-methylbutyl cyanide, n-pentyl cyanide, 4-methylpentyl cyanide, n-hexyl cyanide, 3-(methylthio) propyl cyanide, benzyl cyanide, 4-(methylthio) butyl cyanide, and 2-phenylethyl cyanide. Isothiocyanates: allyl thiocyanate, allyl isothiocyanate, 2-methylpropyl isothiocyanate, but-3-enyl isothiocyanate, 3-methylbutyl isothiocyanate, 4-methylpentyl isothiocyanate, 3-(methylthio)propyl isothiocyanate, benzyl isothiocyanate, 4-(methylthio) butyl isothiocyanate, 2-phenylethyl isothiocyanate, and 5-(methylthio) pentyl isothiocyanate Aldehydes: hexanal, furfural; 3-(methylthio) propanal, phenylacetaldehyde, nonanal, non-2(E)-enal, deca-2(E),4(E)-dienal, and syringaldehyde The predominant compounds were cyanides: 4-(methylthio) butyl cyanide (61.3%, 66.3%, and 79.6% (*w*/*w*), respectively), 3-(methylthio) propyl cyanide (21.7%, 21.6%, and 10.7% (*w*/*w*)), and isothiocyanates: 4-(methylthio) butyl isothiocyanate (5.3%, 4.0%, and 6.7% (*w*/*w*)) for the three oils	[125]
	*B. tournefortii*	*Gouan*	Volatile oil	diethyl ether	GC–MS	14 compounds (76.1% (*w*/*w*)): propane, 1-isothiocyanato-3-(methylthio) (24.39% (*w*/*w*)), 2-propenal, 3-(2,6,6-trimethyl-1-cyclohexen-1-yl), ionone (1.7% (*w*/*w*)), aromadendrene (6.69% (*w*/*w*)), 2-pentadecanone,6,10,14-trimethyl, elimicin (1.864% (*w*/*w*)), 8,11-octadecanoic acid, methyl ester, α –bisabolol, 1-hexadecanol, hexadecanoic acid, di-2-propenyltetrasulfide (19.803%), n-heneicosane, 12-octadecanoic acid, and n-octadecanoic acid	[126]

## 4. Pharmacological Properties of *Brassica* Genus Seeds

According to the existing literature, *Brassica* genus seeds are full of many biologically active compounds which have been associated with a host of potential health benefits. Indeed, the mustard extract seeds have been linked to several biological properties such as antioxidant, antiproliferative, antimicrobial, antibacterial, antidiabetic, anti-inflammatory, and neuroprotective activities.

### 4.1. Antioxidant Activity

*Brassica* species constitute a source of antioxidant compounds such as α-tocopherol, ascorbic acid, canolol, carotenoids (lutein and β-carotene), phenolic acids (gallic acid, caffeic acid, sinapic acid, ferulic acid, and 3,4-di-hydroxybenzoic acid), and flavonoids (rutin, quercetin, and kaempferol), which can protect the immune system by neutralizing free radicals [6,10,106,127]. Antioxidants, such as phenolic compounds, considered as protective agents, are involved in the adsorption and neutralization of reactive oxygen species (ROS), quenching singlet and triplet oxygen or decomposing peroxides. Many long-term illnesses, including cancer, atherosclerosis, aging, inflammation, and neurological illnesses such as Parkinson’s and Alzheimer’s disease, are directly linked to ROS [128,129]. Additionally, a positive correlation between the antioxidant capacity of *Brassica* species and their profile and content of polyphenols, especially flavonoids, was established because phenolic compounds show higher antioxidant activity than vitamins and carotenoids [6,130]. Sinapic acid, 3,4-di-hydroxybenzoic acid, ferulic acid, and rutin identified from *Brassica* seeds were strongly associated with the antioxidant capability [108]. Quercetin, the major representative of the flavonol subclass, shows antioxidant potential by scavenging free radicals and chelating transition metal ions [131]. Furthermore, kaempferol is also characterized by a remarkable antioxidant potential [132].

Because various oxidants have distinct recovery processes, several assays are employed to assess the total antioxidant capacity of samples. The seeds have a high antioxidant potential, as evaluated by their ability to reduce and chelate metals, reduce lipids, and scavenge free radicals. 2,2-Diphenyl-1-picrylhydrazyl (DPPH) radical-scavenging activity and the ferric reducing antioxidant power (FRAP) are the most widely used tests for evaluation of the antioxidant capacity of plant samples. The antioxidant activities of different mustard seeds as reported in the relevant literature are summarized in Table 5. In vitro antioxidant activity has been the subject of numerous studies for different *Brassica* species seeds. Several reports have highlighted the strong antioxidant potential of seeds in terms of positive DPPH radical-scavenging activity, Fe^2^⁺-chelating effect (FRAP), oxygen radical absorbance capacity (ORAC), and [2,2′-azinobis-(3-ethylbenzothiazoline-6-sulfonic acid)] (ABTS) radical-cation-scavenging activity. As indicated in Table 5, the cold-press oil of *Sinapis alba* (white mustard) and *B. nigra* (black mustard) seed extract are characterized by a higher inhibition of DPPH with 94.24% and 89.25%, respectively [78], followed by *B. juncea* with 83.17% [81]. The antioxidant potency of *B. juncea* seeds has been largely reported [62,81,133,134,135]. Furthermore, it has been reported that the dichloromethane extract of *B. rapa L.* (Turnip) seeds exhibited a potent in vivo antioxidant effect through the inhibition of the hydroxyl radicals responsible for oxidative damage to DNA at 1000 μg/mL concentration by phenylethylbrassinin and 3-phenylpropionitrile [123]. Recently, special emphasis was placed on the antioxidant potential of broccoli (*B. oleracea. var. italica*), especially its seeds [77,136] and their isolated compounds such as sulforaphane [79]. The antioxidant potential of different *Brassica* species seeds and their bioactive compounds was evaluated in animals to better highlight and understand their mechanism action. An in vivo investigation on female albino rats exposed to cadmium chloride, CdCl_2_, showed that the aqueous extract of *B. nigra* seeds prevented the oxidative stress of cadmium chloride and its toxicity in hematological parameters and lung tissue [137]. Moreover, a mustard-seed-enriched diet improved the antioxidant status in mice by increasing plasma antioxidant enzyme activity and dose-dependently reducing lipid peroxidation [138]. Moreover, the administration of broccoli and seed-purified glucoraphanin proved effective to reduce oxidative stress promoted by a high-fat diet (HFD) in mice by raising superoxide dismutase (SOD) and catalase (CAT) activities [139]. The marked antioxidant and antigenotoxic status of *B. juncea* seeds and their nanoparticles were demonstrated in an in vivo male Wistar rat model against metal toxicity, particularly arsenic, responsible for oxidative damage in the brain [140].

In summary, *Brassica* spp. seeds and their bioactive compounds have exhibited a strong antioxidant effect in both in vitro and in vivo experiments, indicating their potential application in antioxidant treatments of chronic disorders induced by ROS.

### 4.2. Cytotoxic Activity

The drugs used for cancer therapy are toxic and affect both cancer cells and normal cells. Hence, it is necessary to use compounds isolated from natural sources to reduce cancer risk in several cancer types, including colon cancer, lung cancer, gastric cancer, and breast cancer. The significant antiproliferative and preventative effect of *Brassica* vegetables seeds on tumor cells, most consistently colon and lung cancers [143,144], are related to their richness in bioactive components such as phenolics, flavonoids, glucosinolates, and their degradation products allyl isothiocyanate acid, sulforaphane, and indole-3-methanol. In addition to their antioxidant capacity, phenolics and flavonoids have been found to possess antitumor activity [145,146]. Quercetin and kaempferol have been shown to inhibit cell growth in human intestinal cancer lines in a synergistic manner [147]. In addition, quercetin and rutin have shown antiproliferative activity against largeous spectrum of different cancers [148,149]. It has also been reported that sinigrin inhibits the proliferation of breast cancer cells [150] and liver cancer cells [151]. As it is known, glucosinolates and their degradation products such as allyl isothiocyanate, benzyl isothiocyanate, phenethylnate, and sulforaphane inhibit the activity of various cancer cells [152,153,154,155,156]. Sulforaphane, 3,3′-diindolylmethane, and indole-3-carbinol inhibit several carcinoma cells by inhibiting the transcription factors (STAT) and by suppressing cyclin-dependent kinase CDK2/4/6, cyclin D, and P27Kip expression [157,158,159].

The reported anticarcinogenic activity of mustard seeds is shown in Table 6. Compared to other *Brassica* seeds, the oil seed of *B. juncea* var. *raya* revealed a remarkable anti-proliferative activity against a large spectrum of different human cancer cell lines, mainly breast (MCF-7 and MDA-MB-231), prostate (PC-3), lung (A-549), cervix (HeLa), and colon (HCT-116) with a high inhibition in MCF-7 cells with an IC_50_=32.93 μg/mL. It was proven that seed oil reduced the increase in cancer cells in a dose-dependent manner, resulting in apoptosis and cell death [97]. Another study in *B. oleracea var. capitata* seed fractions revealed moderate anticancer activities of fractions II (IC_50_=27.32 μg/mL) and III (IC_50_=15.56 μg/mL) on A-549 cells compared to those of sulforaphane (IC_50_=3.53 μg/mL) > iberin (IC_50_=4.93 μg/mL) > iberverin (IC_50_=7.07 μg/mL). They also induced cell apoptosis by increasing early apoptosis and late apoptosis/necrosis and activating caspase-3, -8, and -9 [160]. Hexane, ethyl acetate, ethanol, and methanol extracts of broccoli seeds exhibited high anticancer activity on A-549, LAC, HeLa, HepG-2, and Caco-2 with a high inhibition of LAC with IC_50_ of 10.38 mg/g [63,77]. However, the essential oil extracted using diethyl ether from *B. tournefortii* seeds showed the highest antiproliferative activity against MCF-7 (IC_50_=1.34 µg/mL) and HCT-116 (IC_50_=4.5 µg/mL) [126]. Furthermore, the cytotoxic activity of the ethalonic extract of *B. nigra* seeds was exerted via the reduction in viability and clonogenic survival of A-549 and H-1299 cells, the induction of cellular apoptosis in a time- and concentration-dependent manner, and the increase in caspase-3 activity [161]. Canolol at 100 μM concentration, isolated from rapeseed oil, reduced human colon cancer cell line (SW480) apoptotic death, and it was also found to be an effective anticarcinogenic chemical in the *Salmonella typhimurium* modified Ames assay [162]. In addition, mustard seed emulsion was found to be able to attenuate 1,2-dimethylhydrazine (DMH) and azoxymethane (AOM)-induced mice colon carcinogenesis, by reducing growth and activating the apoptotic death of (SW480) [138,163]. At a concentration of 800 mg/kg, the isothiocynate-rich hydro-alcoholic extract of *B. nigra* seeds exhibited an antiproliferative effect on the liver tissue of phenobarbital-exposed mice by ameliorating histopathological changes including moderate diffuse proliferation and eosinophilic cytoplasm [164].

### 4.3. Antimicrobial Activity

In addition to their agricultural and nutritional importance, numerous studies have highlighted the appreciable antimicrobial and fungicidal potential of *Brassica* seeds against various important pathogens, as they are a predominant source of proteins, polyphenols, glucosinolates, and essential oils [6]. RCN seed meal of *B. rapa* showed a large antimicrobial spectrum, mainly against food-borne pathogens, Gram-positive bacteria (*Bacillus cereus* (minimum inhibitory concentration (MIC)=32 µg/mL), *Staphylococcus aureus* (MIC=32 µg/mL), *Enterococcus faecalis* (MIC=16 µg/mL), and *Listeria monocytogenes* (MIC=128 µg/mL)), Gram-negative bacteria (*Escherichia coli* (MIC=256 µg/mL), *Proteus mirabilis* (MIC=128 µg/mL), *Proteus vulgaris* (MIC=128 µg/mL), *Pseudomonas aeruginosa* (MIC=256 µg/mL), *Salmonella typhi* (MIC=128 µg/mL), *Enterobacter cloaceae* (MIC=64 µg/mL), *Yersinia enterocolitica* (MIC=64 µg/mL), and *Klebsiella pneumoniae* (MIC=128 µg/mL)), yeasts (*Candida albicans 10231* (MIC=64 µg/mL), and *Candida albicans 90028* (MIC=64 µg/mL)), and molds (*Fusarium oxysporum* (MIC=128 µg/mL), *Cladosporium herbarum* (MIC=128 µg/mL), *Botrytis cinerea* (MIC=64 µg/mL), and *Aspergillus flavus* (MIC=128 µg/mL)). Individually, the best antimicrobial activity of RCN seed meal extract was toward *Enterococcus faecalis* (MIC=16 µg/mL) and *Staphylococcus aureus* (MIC=32 µg/mL) [51]. However, with *B. rapa* seeds, positive activity was found only against *Plasmodium berghei* infection in mice and *Escherichia coli*, *Klebsiella pneumonia*, *Salmonella para-typhi*, *Pseudomonas aeuriginosa*, and *Staphylococcus aureus*, with the inhibition zone diameters ranging between 7 and 23 mm [64,165]. Moreover, it has been reported that ethanol, methanol, and water broccoli seed extracts showed an inhibitory effect against Gram-negative bacteria, e.g., *Staphylococcus aureus* and *Bacillus subtilis*, and Gram-positive bacteria, e.g., *Salmonella typhimurium* and *Escherichia coli* [77].

Isolation of antimicrobial proteins and peptides, identified as APPs, from seeds of *Brassica* species is immensely increased owing to their remarkable antimicrobial activity with various mechanisms of action. These natural chemicals are being studied as anti-infectives [166]. Several AMPs from *Brassica* seeds have been isolated with promising antimicrobial potential against numerous pathogens responsible for microbial infections. Indeed, the 8.5 kDa purified antifungal peptide, BGAP, from the crude extract of *B. oleracea* var. *gongylodes* seeds demonstrated a large antifungal spectrum, mainly against *Colletotrichum higginsianum* (IC_50_=17.33 μg/mL), *Exserohilum turcicum* (IC_50_=12.37 μg/mL), *Magnaporthe oryzae* (IC_50_=16.81 μg/mL), and *Mycosphaerella arachidicola* (IC_50_=5.60 μg/mL) [167]. Another study found that broccoli seed peptides BraDef1 and BraDef2, identified as class I definsins, provided antimicrobial properties against fungi such as *Colletotrichum gloeosporioides* and *Alternaria alternata* and against pathogenic bacteria strains such as *Bacillus cereus 183*, *Pseudomonas aeruginosa*, *Listeria monocytogenes*, *Vibrio parahaemolyticus ATCC 17,802,* and *Salmonella typhimurium* [168]. Furthermore, a novel antifungal protein (15 kDa), a napin-like protein named broccoli napin (BoNap), was isolated from *B. oleracea L*. var. *italica* and shown to be responsible for blocking the germination of *Fusarium culmorum* and *Penicillium expansum*’s spores with a minimum inhibitory concentration (MIC) value of 2.31 µΜ. Moreover, BoNap was found to induce the permeabilization of mycelium membrane, trypsin-inhibitory properties, and the reduction of fungal contamination of cereals [169]. *Allergen Sin a1*, the 14 kDa mustard Napin protein purified from *B. hirta* seeds (white mustard), was determined as a potent bioactive agent against a broad spectrum of microbial strains, especially *Saccharomyces cerevisiae DSM 70449*, *Zygosaccharomyces bailii Sa 1403,* and Fusarium *culmorum FST 4.05*, with an MIC range between 3 μM and 6 μM, and with a noncytotoxic effect in mammalian cells. *Sin a1* is a stable protein, resistant to α-chymotrypsin digestion, high temperature, pH changes, and salts [170]. The purified peptide, *γ-thionin* (BoT), from *B. oleracea* seeds was reported as a potent antifungal agent against *Aspergillus niger* and *Aspergillus flavus* at 2 μM concentration and led to the death of the insects *Tribolium castaneum* and *Sitophilus oryzae* at 0.12 μM concentration [171].

Beyond their anti-cancer potential, glucosinolates, especially sinigrin (prop-2-enylglucosinolate), glucotropeolin (benzylglucosinolate), and gluconasturtiin (phenethylglucosinolate), isolated from mustard seed meal, are characterized as potent bioactive agents against different fungi such as *Botrytis cinerea*, *Fusarium oxysporum*, *Aphanomyces euteiches var. pisi*, *Pseudocercosporella herpotricoides*, *Rhizoctonia solani*, *Gaeumannomyces graminis var. tritici,* and *Verticillium dahlia* [172]. Furthermore, purified sulforaphane from broccoli seeds had a significant effect on *Escherichia coli ATCC 25922* growth at low levels (5–25 μg/mL), whereas levels of sulforaphane exceeding 200 µg/mL may cause sigmoid growth kinetics deform [173]. 

Moreover, recent clinical trials on *SARS-CoV-2* patients proved that the intake of broccoli seed capsules with glucoraphanin and myrosinase reduced COVID-19 symptoms for 6 to 12 h as a function of the *Nrf2*-interacting nutrients [174].

Collectively, these research data suggest that *Brassica* seeds and their derived compounds (e.g., functional peptides, GLS, and sulforaphane) could be exploited as natural antimicrobials in the food and pharmaceutical sectors.

### 4.4. Antidiabetic Activity

Diabetes is one of the major diseases causing morbidity and mortality worldwide. Several research studies have demonstrated the potential hypoglycemic effect of seeds of the *Brassica* genus. *Brassica* seed extracts have been tested in vitro and in animals. The *B. juncea* seed diet (10%, 15%) showed potent antihyperglycemic effects in alloxan diabetic rats, whereas daily oral feeding of 10% powder of seeds of *B. juncea* for 60 days showed weak antihyperglycemic activity in a severe hyperglycemic state in streptozotocin (STZ) diabetic rats [175,176]. Another in vivo study of aqueous seed extract of *B. juncea* (250, 350, 450 mg/kg) proved that the significant hypoglycemic activity on STZ-induced diabetic male albino rats was related to the presence of isothiocyanate glycoside sinigrin, protein, and fixed oil [177]. Moreover, the administration of oil purified from *B. nigra* seeds at a dose of 500 and 1000 mg/kg body weight to STZ diabetic rats caused a reduction in blood glucose level of STZ diabetic rats from 335 to 280 mg/dL and from 330 to 265 mg/dL at 4 h, respectively, as compared with the diabetic control, along with a significant increase in body weight, liver glycogen content, and plasma insulin level, as well as a decrease in glycosylated hemoglobin, a decrease in malondialdehyde (MDA), and an increase in reduced glutathione (GSH) in test groups as compared to the control group. The obtained results indicated that the *B. nigra* seed oil at both doses remarkably induced an antihyperglycemic effect [178].

The consumption of padding enriched with soluble dietary fiber (DF) from mustard seed mucilage significantly attenuated the blood glucose and insulin levels at specified post-meal intervals in adults with high type 2 diabetes risk [179].

### 4.5. Anti-Inflammatory Activity

In addition to the properties already mentioned, it has been reported that *Brassica* seeds, particularly broccoli seeds, showed a stronger anti-inflammatory effect higher than carrot and cucumber seeds [136]. Furthermore, *Sinapis alba* seeds exhibited a significant inhibition of the mouse prostatic hyperplasia induced by testosterone propionate and the serum acid phosphatase activity by sinalbin and beta-sitosterol (16.0 and 8.0 mg/kg/day). Sinalbin and beta-sitosterol have anti-androgen and anti-inflammatory activities [180,181]. 

### 4.6. Regulation of Metabolic Syndrome

Hypertension, obesity, T2D, and dyslipidemia are common metabolic syndrome disorders prevalent worldwide [182]. The regulatory effects of *Brassica* seeds and their derivatives on metabolic syndrome have been confirmed in numerous studies.

The seed glucoraphanin diet modulated obesity in HFD-fed mice by regulating lipid metabolism genes and increasing the abundance and diversity of the gut microbiota [139].

Cold-press rapeseed oil preserved a high level of bioactive compounds (MUFA, PUFA, tocopherols, and phytosterols) that were positively correlated with numerous health benefits, such as hyperlipidemia regulation [183]. Its consumption considerably lessened the oxidative stress, the total cholesterol, oxidized LDL, and LDL cholesterol levels among metabolic syndrome-afflicted men [184]. Additionally, the oral treatment of melatonin-rich mustard seeds in rats downregulated de novo cholesterol production by inhibiting the activity of hepatic 3-hydroxy-3-methyl-glutaryl-CoA (HMG-CoA) reductase and scavenging the hypercholesterolemia-related ROS [185]. Overdosing on paracetamol acetaminophen (APAP) damaged the liver tissue, whereas the in vitro intervention of the extracted antioxidant-rich fraction from mustard seeds and their isolated phytoconstituents (quercetin, catechin, and vitamin E) successfully restored the APAP-induced toxicity in a hepatocellular carcinoma (HepG2) cell line. The hepatoprotective potential of the hydromethanolic mustard seed extract was linked to its ability to maintain the hepatocyte membrane integrity by blocking radical–macromolecule binding and reducing hepatic enzymes levels to normal values [62].

### 4.7. Neuroprotective Activity

Psychological and neurodegenerative disorders include depression, anxiety, autism spectrum disorder (ASD), Alzheimer’s, Huntington’s, Parkinson’s, and schizophrenia which exhibit oxidative stress, mitochondrial dysfunction, inflammation, and neural damage [186]. Current research has proven the neuroprotective benefits of *Brassica* seeds and their derivatives in vitro and in humans. The new nitrogenous compound, brassicalkaloid A, and the known alkaloid coixspirolactam C, isolated from *B. napus* L. seeds, displayed an anti-neuroinflammatory effect by suppressing the nitric oxide (NO) production induced by lipopolysaccharide (LPS) in BV-2 cells with IC_50_ = 36.6 and IC_50_ = 51.0 μM respectively, compared to the positive control NG-monomethyl-L-arginine (L-NMMA) (IC_50_ = 17.4 μM) [102]. A recent clinical study on autism spectrum disorder (ASD) children revealed that the consumption of high-sulforaphane broccoli seed and sprout tablets improved their behavior and social responsiveness and identified changes in urinary metabolites correlated with clinical improvements. Consequently, sulforaphane’s neuroprotective effect probably stemmed from transcription factor (Nrf2) activation [187].

*Brassica* seeds and their derived compounds have proven several health benefits in vitro, in vivo, and in clinical trials. However, additional research is needed to evaluate the potential of other isolated bioactive compounds and to investigate the unrecognized bioactivities and their action mechanisms in vitro and in vivo assays such as cardioprotective, gastroprotective, and renoprotective potentials.

## 5. Toxicological Effects of *Brassica* Seeds

Currently, special emphasis has been placed on the use of *Brassica* seeds in the food and beverage industry, as well as in nonfood uses as they represent a rich source of biologically bioactive compounds, strongly associated with broadly recognized nutritional and functional properties. Among these compounds, erucic acid, glucosinolates, and their degradation products, as well as allergens, have been characterized by undesirable effects on human and animal health, mainly when consumed in high doses and in concentrated or isolated form [188].

Erucic acid or cis-13-docosenoic acid (C22:1) is the predominant monounsaturated fatty acid in *Brassica* seeds. The intake of high amounts of erucic acid damages the liver, kidneys, muscles, and testes. In animal trials, the heart is the most negatively affected by erucic acid’s harmful effect after either brief or prolonged exposure due to the accumulation of triacylglycerol (TAG), known as myocardial lipidosis. In 2016, the EFSA (European Food Safety Authority) set an acceptable daily intake (ADI) of 7 mg/kg body weight [189], whereas, according to the Food Standards Code of Australia and New Zealand (FSANZ) and the Commission Regulation (EU) 2019/1870, a maximum amount of 20 g/kg (2%) in edible oils is imposed [190,191]. To ensure the safety of oils for both human and livestock consumption, antinutritional components of the seed have to be removed using the conventional breeding process through the development of canola (1970). Canola edible oil seed, with low erucic acid (less than 2%) and low aliphatic glucosinolate (less than 25 µmol/g), is considered one of the most popular healthy cooking oils for its low saturated fatty acid content, high monounsaturated fatty acids, and balanced content of omega-3 fatty acids. Additionally, to raise the nutritional quality and quantity of oil from *B. napus* L., different modifications involving biotechnology, agronomy, genetic interventions, and bioengineering processes have been applied [192]. Despite the toxic effects of erucic acid intake on human and animal health, it is used in a diverse range of nonfood applications, including cosmetics, medicines, plasticizers, and detergents. *Brassica* oils with high erucic acid levels exceeding 55% still receive interest for application in industry processes [193]. In particular, to produce seeds with high oil for industrial uses, genetic studies of candidate genes and their regulatory mechanisms are being developed [194,195].

*Brassica* seeds have abundant proteins with a predominance of napin and cruciferin seed storage proteins, identified as major allergens in humans [59]. Despite the positive nutritional and antimicrobial effects of the proteins isolated from *Brassica* seeds, it has been proven in numerous clinical trials and case reports that the intake of mustard seeds or products made from them, even in small amounts, immediately causes severe systemic reactions, including anaphylaxis [196]. With the lack of effective preventive treatment for mustard allergy, informative labeling remains the only solution for allergy prevention in children and consumers allergic to mustard protein.

Glucosinolates, mustard oil glucosides, are one of the most relevant biomolecules of *Brassica* vegetables, responsible for their bitter and pungent taste. GLS content and profile differ by *Brassica* species and organ, with a high amount in seeds. Generally, *B. nigra* (L.) *W. D. J. Koch* was declared to have the highest GLS content followed by *B. oleracea Alboglabra* with 201.95  and 180.9 µmol/g, respectively [197]. Interestingly, the intake of *Brassica* vegetables and seeds is closely associated with a wide range of therapeutic properties in the prevention and treatment of several chronic diseases, especially cancer risks due to the richness in GLS and their breakdown products [97,153,155,164]. Conversely, the intake of *Brassica* plants caused death [198] and liver disease in animals [199]. Similarly, the consumption of de-oiled seeds with excess amounts of hydrophilic glucosinolates is deleterious to domestic animals, according to numerous studies [200]. This is mainly due to the accumulation of progoitrin and sinigrin in *Brassica* seeds after oil extraction, leading to a bitter aftertaste responsible for reduced dietary intake [201]. Intact GLSs are nontoxic, while the degradation products generated after myrosinase hydrolysis are more toxic. It has been shown that nitrile progoitrin with 2–3 mmol/kg induced liver, pancreatic, and kidney toxicity in rats [198], and the progoitrin breakdown generates goitrogenic compounds [200]. The principal toxic consequences in animals that have been documented are fetal death in mammals, decreased avian egg production, liver and kidney hypertrophy, and thyroid gland modification [202]. Typically, these harmful and antinutritional effects appear mostly in animal species rather than in humans at high doses. Thus, the removal of maximum GLS without coextraction of proteins in oil-extracted meal was required in animal feed [203]. Furthermore, thiocyanate ions and axazolidin-2-thiones formed from GLS are goitrogenic and cause thyroid cancer [204]. It was proven that *Brassica* vegetable intake was linked to thyroid cancer in a study on 293 women [205]. The ADI set by EFSA is 20 g/kg of body weight, while AITC’s daily intake should be 1 mg without exceeding the ADI of 1.4 mg for an adult of 70 kg of body weight [206]. Therefore, in order to reduce the seed GSL content (SGC) in mature seeds to increase their economic and nutritional value, research has been directed toward the study of the genetic structure of SGC to deeply understand the regulatory genes and their genetic mechanisms controlling both seed GSL synthesis and accumulation [207].

## 6. Conclusions

Black mustard (*B. nigra*), *B. oleracea*, rapeseed (*B. rapa*), Ethiopian mustard (*B. carinata*), Asian mustard (*B. juncea*), oilseed rape (*B. napus*), African mustard (*B. tournefortii*), and canola plants are nutritionally, economically, medicinally, and pharmaceutically important in the world. They are the major economically important oilseed crops in many countries. *Brassica* seeds have been employed since antiquity as an oil source, as a food condiment, in traditional medicine, and as animal feed. Recently, they have gained increasing interest for the extraction and characterization of their health-promoting compounds. In this review, we summarized the research data on the chemical composition, pharmacological proprieties, and toxicological effects of *Brassica* spp. seeds and their derivatives. Research has proven their richness in proteins, minerals, vitamins, phenolic compounds, GLS, and carotenoids with a variable profile. Several phytochemical compounds belonging to this group have been identified and quantified, but data remain limited, and further investigations are needed. The bioactive compounds of *Brassica* seeds (proteins, polyphenols, GLS, carotenoids, fatty acids, and alkaloids) are responsible for different medicinally significant pharmacological properties such as antioxidant, anticancer, antibacterial, antifungal, antidiabetic, and neuroprotective activities, as well as in metabolic disorder regulation. Due to their wide range of benefits, *Brassica seeds* show a significant opportunity for the development of natural antioxidant products and drugs. Further research is needed to investigate the potential of isolated and purified bioactive compounds for use in the food industry to enrich and improve food quality and in clinical interventions in the field of human health, e.g., to increase the activity of established treatments for cancer, diabetic, and metabolic syndrome diseases, taking into account the health toxicity of seed compounds, such as glucosinolates and erucic acid, in clinical trials. 

## Figures and Tables

**Table 1:** Diversity of the genus *Brassica* species [10].

Species	Subspecies/var.	Common Name
*Brassica nigra*	*Koch* L.	Black mustard
	*Viridis*	Collards
*Brassica oleracea*	*Capitata F. alba*	White cabbage
	*Capitata F. rubra*	Red or purple cabbage
	*Capitata* L.	Green cabbage
	*Italica*	Italian broccoli, Chinese broccoli
	*Gemmifera*	Brussels sprouts
	*Sabellica* L.	Curly kale
	*Acephala* L.	Kale
	*Alboglabra*	Chinese kale, kailan
	*Botrytis*	Cauliflower, Italian cauliflower
	*Sabauda*	Savoy cabbage
	*Gongylodes*	Kohlrabi, stem turnip, Knol khol
	*Costata*	Portuguese cole, Tronchuda cabbage
*Brassica carinata*		Ethiopian rapeseed
*Brassica juncea*	*Czern* L.	Mustard, Indian mustard, leaf mustard
	*Coss* L.,	Green mustard
	*Integrifolia*	Korean leaf mustard, multi-shoot mustard
	*Crispifolia*	Curled mustard
	*Rosularis*	Tatsoi
*Brassica napus*	*Napobrassica*	Oilseed rape, rape, canola
*Brassica hirta*	*Sinapis alba*	White or yellow mustard
*Brassica tournefortii*	*Gouan*	Asian mustard, Africain mustard
*Brassica rapa/campestris*	*Rapifera* L./*Rapa* L.	Sarson, turnip rape, field mustard, bird, rape, canola, turnip top
	*Pekinensis* L.	Chinese cabbage
	*Parachinesis*	Chines cabbage, Choi sum, Sawi

**Table 2:** Mean composition of oil content and main fatty acids (%).

		Fatty Acid Composition in the Oil							
Species	Oil Content	Palmitic Acid	Stearic Acid	Oleic Acid	Eicosenoic Acid	Linoleic Acid	Linolenic Acid	Erucic Acid	Reference
*B. nigra*	37.68	3.16	1.41	27.1	6.83	14.87	7.98	32.96	[12,34]
*B. oleracea* L. var. *acephala*				10		10	10	>50	[35]
*B. oleracea.* L. var. *italica* cv. *Legacy*		7.43	1.10	15.44	4.64	18.15	12.37	9.36	[36]
*B. rapa*	47.3			12	8–9	13	8–9	50–51	[37]
*B. carinata*	40								[38]
*B. napus*	42.8; 46.4			12	8–9	13	8–9	42–54	[37,39]
*B. hirta*		14.55		9.67					[40]
*Sinapis alba*								23.90	[41]

**Table 5:** Study on antioxidant activity of different Brassica species seeds.

Species	Subspecies/var	Sample Analyzed	Extracting Solvent	DPPH	FRAP	ORAC	ABTS	ROS	Reducing Power	SOA	Hydroxyl Radical	Reference
*B. nigra*		Cold-press oil	Ethanol	89.25% (10% oil–methanol)	23.85% (10% oil–methanol)							[78]
		Seeds	Hexane, ethyl acetate, and methanol	36.30% (1 mL of crude extracts)								[63]
*B. oleracea*	*L.* var. *costata DC*	Lyophilized extract	H_2_O							IC25 at 197 µg/mL	IC25 = 4 µg/mL	[103]
	*italica*	5 day old sprout (PS5) Seeds	H_2_O							94.25% (2 mg/mL)		[141]
		Seeds (USA)	50% acetone			633.50 μM TE/g (133 mg/mL)	175.88 μM TE/g (133 mg/mL)					[136]
	*botrytis*	5 day sprouts (C5D) 7 day sprouts (C7D)	DCM	62.2%; 39.63% (2 mg/mL)								[134]
				IC_50_ = 1510 µg/mL, IC_50_ = 2750 µg/mL						IC_50_ = 170 µg/mL, IC_50_ = 260 µg/mL		
		Seeds	80% methanol	70% (25 µg/mL)								[142]
*B. rapa*	*RCN*	Seed meal	methanol	2947.2 µmol/L (10 mg/L)	1852.1 µmol/L (10 mg/L)							[51]
	*Rapifera* L.	(T5D) (T7D)	DCM	21.98%; 40.45% (2 mg/mL)								[134]
				IC_50_ = 5920 µg/mL, IC_50_ = 2780 µg/mL						IC_50_ = 53 µg/mL, IC_50_ = 0.32 µg/mL		
*B. juncea*		Seeds	80% alcohol	IC_50_ = 103 µg/mL								[133]
	L. Czern and Coss	Seeds	Hydromethanol (80:20 ratio)	IC_50_ = 103.37 µg AAE/mg	IC_50_ = 83.26 µg AAE/mg	IC_50_ = 1115 µM GAE/mL	IC_50_ = 83.05 µg GAE/mL	Post-treatment (700 µg/mL): 58.37 to 15.55% Pretreatment (1000 µg/mL): 90.5%		IC_50_ = 345.22 µg AAE/mg		[62]
		(M5D)/(M7D)	DCM	40.78%; 19.67% (2 mg/mL)								[134]
				IC_50_ = 2760 µg/mL, IC_50_ = 5790 µg/mL						IC_50_ = 59 µg/mL, IC_50_ = 463 µg/mL		
		Seeds	Ethanol	83.17% (200 mg/mL)			60.57% (200 mg/mL)					[81]
		Seeds	30% ethanol, water	IC_50_ = 170 µg/mL, IC_50_ = 390 µg/mL			75.5% 68.9% (50 mg/mL)					[120]
*B. napus*	*Canola*	Defatted oilseed cake	Methanol/acetone/water (MAW)	33.03% (60 mg/mL)	8.78 μmol Fe(II)/g FW (60 mg/mL)							[83]
*B. hirta*		Seeds	H_2_O						6.4 mg/g (75 mg/mL)			[76]
	*Sinapis alba*	Cold-press oil	Ethanol	94.24% (10% oil–methanol)	8.92% (10% oil–methanol)							[78]
*B. tournefortii*	*Gouan*	Essential oil	Diethyl ether	45.17 vitamin C (IC_50_ = 75.28 µg/mL)								[126]

IC_50_: inhibitory concentration required for 50% inhibition, TE: Trolox equivalent.

**Table 6:** Antiproliferative activity of the *Brassica* seed extracts IC_50_ (µg/mL).

Species	Subspecies/var	Sample Analyzed	Extracting Solvent	A-549	MCF-7	MDA-MB-231	PC-3	HeLa	HCT-116	LAC	HepG-2	Reference
*B. oleracea*	*capitata*	ITCs: fraction II	Ethyl acetate, hexane	27.32	23.85% (10% oil–methanol)							[160]
		ITCs: fraction III		15.56								
	*italica (broccoli)*	Seeds	Hexane, ethyl acetate, methanol	14.38 mg/g				19.45 mg/g		10.38 mg/g	26.75 mg/g	[96]
	*botrytis*	Sprouts C5D; C7D	DCM				95.57, 71.58					[134]
*B. rapa*	*Rapifera* L.						63.5, 43.61					
*B. juncea*	*raya*	Volatile oil	Ethyl acetate, DCM		32.93	37.16	54.73	67.25	61.50			[97]
	*L. Czern*	M5D; M7D	DCM				111.6, 81.11					[134]
*B. tournefortii*	*Gouan*	Essential oil	Diethyl ether		1.34				4.5			[126]

## Data Availability

Not applicable.

## References

[1] Shankar S., Segaran G., Sundar R.D.V., Settu S., Sathiavelu M. (2019). Brassicaceae-A Classical Review on Its Pharmacological Activities. Int. J. Pharm. Sci. Rev. Res..

[2] Ramirez D., Abellán-Victorio A., Beretta V., Camargo A., Moreno D.A. (2020). Functional Ingredients from Brassicaceae Species: Overview and Perspectives. Int. J. Mol. Sci..

[3] Peña M., Guzmán A., Martínez R., Mesas C., Prados J., Porres J.M., Melguizo C. (2022). Preventive Effects of Brassicaceae Family for Colon Cancer Prevention: A Focus on in Vitro Studies. Biomed. Pharmacother..

[4] da Mattosinhos P.S., Sarandy M.M., Novaes R.D., Esposito D., Gonçalves R.V. (2022). Anti-Inflammatory, Antioxidant, and Skin Regenerative Potential of Secondary Metabolites from Plants of the Brassicaceae Family: A Systematic Review of in Vitro and In Vivo Preclinical Evidence (Biological Activities Brassicaceae Skin Diseases). Antioxidants.

[5] El-Esawi M.A. (2015). Taxonomic Relationships and Biochemical Genetic Characterization of Brassica Resources: Towards a Recent Platform for Germplasm Improvement and Utilization. Annu. Res. Rev. Biol..

[6] Salehi B., Quispe C., Butnariu M., Sarac I., Marmouzi I., Kamle M., Tripathi V., Kumar P., Bouyahya A., Capanoglu E. (2021). Phytotherapy and Food Applications from Brassica Genus. Phytother. Res..

[7] Dixon G.R. (2006). Origins and Diversity of Brassica and Its Relatives. Vegetable Brassicas and Related Crucifers.

[8] Šamec D., Pavlović I., Salopek-Sondi B. (2017). White Cabbage (*Brassica oleracea* Var. Capitata f. Alba): Botanical, Phytochemical and Pharmacological Overview. Phytochem. Rev..

[9] Chalhoub B., Denoeud F., Liu S., Parkin I.A.P., Tang H., Wang X., Chiquet J., Belcram H., Tong C., Samans B. (2014). Early Allopolyploid Evolution in the Post-Neolithic Brassica Napus Oilseed Genome. Science.

[10] Nawaz H., Shad M.A., Muzaffar S. (2018). Phytochemical Composition and Antioxidant Potential of Brassica. Brassica Germplasm Charact. Breed. Util..

[11] Favela-González K.M., Hernández-Almanza A.Y., De la Fuente-Salcido N.M. (2020). The Value of Bioactive Compounds of Cruciferous Vegetables (*Brassica*) as Antimicrobials and Antioxidants: A Review. J. Food Biochem..

[12] De Zoysa H.K.S., Waisundara V.Y. (2021). Mustard (Brassica Nigra) Seed. Oilseeds: Health Attributes and Food Applications.

[13] Li H., Xia Y., Liu H.-Y., Guo H., He X.-Q., Liu Y., Wu D.-T., Mai Y.-H., Li H.-B., Zou L. (2021). Nutritional Values, Beneficial Effects, and Food Applications of Broccoli (*Brassica oleracea* Var. Italica Plenck). Trends. Food Sci. Technol..

[14] Jan S.A., Shinwari Z.K., Malik M., Ilyas M. (2018). Antioxidant and Anticancer Activities of Brassica Rapa: A Review. MOJ Biol. Med..

[15] Rahman M., Khatun A., Liu L., Barkla B.J. (2018). Brassicaceae Mustards: Traditional and Agronomic Uses in Australia and New Zealand. Molecules.

[16] Tian Y., Deng F. (2020). Phytochemistry and Biological Activity of Mustard (*Brassica juncea*): A Review. CYTA J. Food..

[17] Rai P.K., Yadav P., Kumar A., Sharma A., Kumar V., Rai P. (2022). Brassica Juncea: A Crop for Food and Health. The Brassica juncea Genome.

[18] Chmielewska A., Kozłowska M., Rachwał D., Wnukowski P., Amarowicz R., Nebesny E., Rosicka-Kaczmarek J. (2021). Canola/Rapeseed Protein–Nutritional Value, Functionality and Food Application: A Review. Crit. Rev. Food Sci. Nut..

[19] Raboanatahiry N., Li H., Yu L., Li M. (2021). Rapeseed (*Brassica napus*): Processing, Utilization, and Genetic Improvement. Agronomy.

[20] USDA-FAS (Foreign Agricultural Service) (2021). Oilseeds: World Markets and Trade.

[21] Food and Agriculture Organization (FAO). FAOSTAT (2021). Statistical Database of the United Nation Food and Agriculture Organization (FAO) Statistical Division.

[22] Han J., Lu C., Li Y., Deng Z., Fu B., Geng Z. (2016). Discrimination of Rapeseeds (*Brassica napus* L.) Based on the Content of Erucic Acid by 1H NMR. Eur. Food Res. Technol..

[23] Lozano-Baena M.-D., Tasset I., Obregón-Cano S., de Haro-Bailon A., Muñoz-Serrano A., Alonso-Moraga Á. (2015). Antigenotoxicity and Tumor Growing Inhibition by Leafy Brassica Carinata and Sinigrin. Molecules.

[24] Aydin S. (2020). Total Phenolic Content, Antioxidant, Antibacterial and Antifungal Activities, FT-IR Analyses of *Brassica oleracea* L. Var. Acephala and Ornithogalum umbellatum L. Genetika.

[25] Szőllősi R. (2020). Indian Mustard (*Brassica juncea* L.) Seeds in Health. Nuts Seeds Health Dis. Prev..

[26] Kasprzak M.M., Houdijk J.G.M., Liddell S., Davis K., Olukosi O.A., Kightley S., White G.A., Wiseman J. (2017). Rapeseed Napin and Cruciferin Are Readily Digested by Poultry. J Anim. Physiol. Anim. Nutr..

[27] Lakwani M.A.S., Kenanoğlu O.N., Taştan Y., Bilen S. (2022). Effects of Black Mustard (*Brassica nigra*) Seed Oil on Growth Performance, Digestive Enzyme Activities and Immune Responses in Rainbow Trout (*Oncorhynchus mykiss*). Aquac. Res..

[28] Kayacetin F. (2020). Botanical Characteristics, Potential Uses, and Cultivation Possibilities of Mustards in Turkey: A Review. Turk. J. Bot..

[29] Balesh T., Zapata F., Aune J.B. (2005). Evaluation of Mustard Meal as Organic Fertiliser on Tef (Eragrostis Tef (Zucc) Trotter) under Field and Greenhouse Conditions. Nutr. Cycl. Agroecosyst..

[30] Monaci E., Casucci C., De Bernardi A., Marini E., Landi L., Toscano G., Romanazzi G., Vischetti C. (2022). *Brassica carinata* Seed Meal as Soil Amendment and Potential Biofumigant. Crops.

[31] Khaliq B., Sarwar H., Akrem A., Azam M., Ali N. (2022). Isolation of Napin from *Brassica nigra* Seeds and Coagulation Activity to Turbid Pond Water. Water Supply.

[32] Kowalski R., Kowalska G., Pankiewicz U., Mazurek A., Włodarczyk-Stasiak M., Sujka M., Wyrostek J. (2019). The Effect of an Addition of Marjoram Oil on Stabilization Fatty Acids Profile of Rapeseed Oil. LWT.

[33] Beyzi E., Gunes A., Beyzi S.B., Konca Y. (2019). Changes in Fatty Acid and Mineral Composition of Rapeseed (*Brassica napus* ssp. *Oleifera* L.) Oil with Seed Sizes. Ind. Crops Prod..

[34] Kaur G., Kaur R., Kaur S. (2022). Studies on Physiochemical Properties of Oil Extracted from *Brassica Nigra* and *Brassica Rapa Toria*. Mater. Today Proc..

[35] Cacciola F., Beccaria M., Oteri M., Utczas M., Giuffrida D., Cicero N., Dugo G., Dugo P., Mondello L. (2016). Chemical Characterisation of Old Cabbage (*Brassica Oleracea* L. Var. *Acephala*) Seed Oil by Liquid Chromatography and Different Spectroscopic Detection Systems. Nat. Prod. Res..

[36] López-Cervantes J., Tirado-Noriega L.G., Sánchez-Machado D.I., Campas-Baypoli O.N., Cantú-Soto E.U., Núñez-Gastélum J.A. (2013). Biochemical Composition of Broccoli Seeds and Sprouts at Different Stages of Seedling Development. Int. J. Food Sci. Technol..

[37] Cartea E., Haro-Bailón D., Padilla G., Obregón-Cano S., del Rio-Celestino M., Ordás A. (2019). Seed Oil Quality of *Brassica napus* and *Brassica rapa* Germplasm from Northwestern Spain. Foods.

[38] Aghdam A.M., Sayfzadeh S., Rad A.H.S., Valadabadi S.A., Zakerin H.R. (2019). The Assessment of Water Stress and Delay Cropping on Quantitative and Qualitative Traits of Rapeseed Genotypes. Ind. Crops Prod..

[39] Slominski B.A., Meng X., Jia W., Nyachoti M., Jones O., Rakow G. Chemical Composition and Nutritive Value of Yellow-Seeded Brassica Napus Canola. Proceedings of the 12th International Rapeseed Congress.

[40] Miyazawa M., Kawata J. (2006). Identification of the Main Aroma Compounds in Dried Seeds of *Brassica hirta*. J. Nat. Med..

[41] Abul-Fadl M.M., El-Badry N., Ammar M.S. (2011). Nutritional and Chemical Evaluation for Two Different Varieties of Mustard Seeds. World Appl. Sci. J..

[42] Stefansson B.R., Hougen F.W. (1964). Selection of Rape Plants (*Brassica napus*) with Seed Oil Practically Free from Erucic Acid. Can. J. Plant Sci..

[43] Quiñones J., Maggiolino A., Bravo S., Muñoz E., Lorenzo J.M., Cancino D., Díaz R., Saenz C., Sepúlveda N., De Palo P. (2019). Effect of Canola Oil on Meat Quality and Fatty Acid Profile of Araucano Creole Lambs during Fattening Period. Anim. Feed Sci. Technol..

[44] Mollers C. Development of High Oleic Acid Oilseed Rape. Proceedings of the 8th International Conference for Renewable Resources and Plant Biotechnology NAROSSA 2002.

[45] Velioglu S.D., Temiz H.T., Ercioglu E., Velioglu H.M., Topcu A., Boyaci I.H. (2017). Use of Raman Spectroscopy for Determining Erucic Acid Content in Canola Oil. Food Chem..

[46] Li J., Liu J., Sun X., Liu Y. (2018). The Mathematical Prediction Model for the Oxidative Stability of Vegetable Oils by the Main Fatty Acids Composition and Thermogravimetric Analysis. LWT.

[47] Amjad Khan W., Chun-Mei H., Khan N., Iqbal A., Lyu S.-W., Shah F. (2017). Bioengineered Plants Can Be a Useful Source of Omega-3 Fatty Acids. Biomed Res. Int..

[48] Alejandre M., Astiasarán I., Ansorena D., Barbut S. (2019). Using Canola Oil Hydrogels and Organogels to Reduce Saturated Animal Fat in Meat Batters. Int. Food Res. J..

[49] Seyyedi S.M., Afshari R.T., Daneshmandi M.S. (2018). The Relationships between Fatty Acids and Heterotrophic Seedling Growth in Winter Canola Cultivars during Accelerated Seed Aging Process. S. Afr. J. Bot..

[50] Radfar M., Rogiewicz A., Slominski B.A. (2017). Chemical Composition and Nutritive Value of Canola-Quality *Brassica juncea* Meal for Poultry and the Effect of Enzyme Supplementation. Anim. Feed Sci. Technol..

[51] Carlo Tenore G., Troisi J., Di Fiore R., Basile A., Novellino E. (2012). Chemical Composition, Antioxidant and Antimicrobial Properties of Rapa Catozza Napoletana (*Brassica Rapa* L. Var. Rapa DC.) Seed Meal, a Promising Protein Source of Campania Region (Southern Italy) Horticultural Germplasm. J. Sci. Food Agric..

[52] Wanasundara J.P.D., McIntosh T.C., Perera S.P., Withana-Gamage T.S., Mitra P. (2016). Canola/Rapeseed Protein-Functionality and Nutrition. OCl.

[53] Hossain Z., Johnson E.N., Wang L., Blackshaw R.E., Gan Y. (2019). Comparative Analysis of Oil and Protein Content and Seed Yield of Five *Brassicaceae* Oilseeds on the Canadian Prairie. Ind. Crops Prod..

[54] Slabas A.R., Harding J., Hellyer A., Roberts P., Bambridge H.E. (1987). Induction, Purification and Characterization of Acyl Carrier Protein from Developing Seeds of Oil Seed Rape (*Brassica napus*). Biochim. Biophys. Acta Lipids Lipid Metab..

[55] Kania J., Gillner D.M. (2016). Characterisation of the Aminopeptidase from Non-Germinated Winter Rape (*Brassica napus* L.) Seeds. Food Chem..

[56] Ascenzi P., Ruoppolo M., Amoresano A., Pucci P., Consonni R., Zetta L., Pascarella S., Bortolotti F., Menegatti E. (1999). Characterization of Low-molecular-mass Trypsin Isoinhibitors from Oil-rape (*Brassica napus* Var. *Oleifera*) Seed. Eur. J. Biochem..

[57] Wanasundara J.P.D. (2011). Proteins of Brassicaceae Oilseeds and Their Potential as a Plant Protein Source. Crit. Rev. Food Sci. Nutr..

[58] Wanasundara J.P.D., Abeysekara S.J., McIntosh T.C., Falk K.C. (2012). Solubility Differences of Major Storage Proteins of *Brassicaceae* Oilseeds. J. Am. Oil Chem. Soc..

[59] Rahman M., Baten A., Mauleon R., King G.J., Liu L., Barkla B.J. (2020). Identification, Characterization and Epitope Mapping of Proteins Encoded by Putative Allergenic Napin Genes from *Brassica Rapa*. Clin. Exp. Allergy..

[60] Rahman M., Guo Q., Baten A., Mauleon R., Khatun A., Liu L., Barkla B.J. (2021). Shotgun Proteomics of *Brassica Rapa* Seed Proteins Identifies Vicilin as a Major Seed Storage Protein in the Mature Seed. PLoS ONE.

[61] Sirvent S., Palomares O., Vereda A., Villalba M., Cuesta-Herranz J., Rodríguez R. (2009). NsLTP and Profilin Are Allergens in Mustard Seeds: Cloning, Sequencing and Recombinant Production of Sin a 3 and Sin a 4. Clin. Exp. Allergy.

[62] Parikh H., Pandita N., Khanna A. (2015). Phytoextract of Indian Mustard Seeds Acts by Suppressing the Generation of ROS against Acetaminophen-Induced Hepatotoxicity in HepG2 Cells. Pharm. Biol..

[63] Syafiqah N., Asnuzilawati A., Syara K., AW N.H., Norhayati Y. (2019). Preliminary Study of Phytochemical Properties & Antioxidant Activities in Seeds of Trigonella Foenum-Graecum, *Brassica Nigra* and *Salvia Hispanica* Species. EAS J. Pharm. Pharmacol..

[64] Danlami U., Orishadipe Abayomi T., Lawal D.R. (2016). Phytochemical, Nutritional and Antimicrobial Evaluations of the Aqueous Extract of *Brassica Nigra* (*Brassicaceae*) Seeds. Am. J. Appl. Chem..

[65] Ogidi O.I., Omu O., Ezeagba P.A. (2019). Ethno Pharmacologically Active Components of *Brassica Juncea* (Brown Mustard) Seeds. Int. J. Pharm. Res. Dev..

[66] Sontakke K.S., Shinde S.L. (2020). Evaluation of the Phytochemical Potential of *Brassica junceal* Seeds. VIIR J..

[67] ALanís-Guzmán M.A., Wesche-Ebeling P., Maiti R. (1995). Chemical, Nutritional and Functional Characterization of Proteins Extracted from Wild Mustard (*Brassica Campestris*, *Brassicaceae*) Seeds from Nuevo Leon, Mexico. Econ. Bot..

[68] Bhandari S.R., Jo J.S., Lee J.G. (2015). Comparison of Glucosinolate Profiles in Different Tissues of Nine Brassica Crops. Molecules.

[69] Hanschen F.S., Schreiner M. (2017). Isothiocyanates, Nitriles, and Epithionitriles from Glucosinolates Are Affected by Genotype and Developmental Stage in *Brassica Oleracea* Varieties. Front. Plant Sci..

[70] Fusari C.M., Nazareno M.A., Locatelli D.A., Fontana A., Beretta V., Camargo A.B. (2020). Phytochemical Profile and Functionality of Brassicaceae Species. Food Biosci..

[71] Altendorf K., Isbell T., Wyse D.L., Anderson J.A. (2019). Significant Variation for Seed Oil Content, Fatty Acid Profile, and Seed Weight in Natural Populations of Field Pennycress (*Thlaspi Arvense* L.). Ind. Crops Prod..

[72] Velasco P., Soengas P., Vilar M., Cartea M.E., del Rio M. (2008). Comparison of Glucosinolate Profiles in Leaf and Seed Tissues of Different *Brassica Napus* Crops. J. Am. Soc. Hortic. Sci..

[73] Oh S., Kim K., Choi M. (2016). Antioxidant Activity of Different Parts of Dolsan Leaf Mustard. Food Sci. Biotechnol..

[74] Abdelazim Mohdaly A.A., Ramadan M.F. (2021). Characteristics, Composition and Functional Properties of Seeds, Seed Cake and Seed Oil from Different *Brassica Carinata* Genotypes. Food Biosci..

[75] Slominski B.A., Jia W., Rogiewicz A., Nyachoti C.M., Hickling D. (2012). Low-Fiber Canola. Part 1. Chemical and Nutritive Composition of the Meal. J. Agric. Food Chem..

[76] Krishnaveni M., Saranya S. (2016). Secondary metabolites, antioxidant activity, phytonutrient analysis of *Nigella sativa* and *Brassica hirta* seeds. Int. J. Pharm. Biol. Sci..

[77] Le T.N., Sakulsataporn N., Chiu C.-H., Hsieh P.-C. (2020). Polyphenolic Profile and Varied Bioactivities of Processed Taiwanese Grown Broccoli: A Comparative Study of Edible and Non-Edible Parts. Pharmaceuticals.

[78] Olgun Ç., Özkan O.E., Güney B., Pattabanoglu E.S., Güney K., Gür M. (2017). Chemical Composition and Antimicrobial Activity in Cold Press Oil of Fennel, Anise, White and Black Mustard Seeds. Indian J. Pharm. Educ. Res..

[79] Lv X., Meng G., Li W., Fan D., Wang X., Espinoza-Pinochet C.A., Cespedes-Acuña C.L. (2020). Sulforaphane and Its Antioxidative Effects in Broccoli Seeds and Sprouts of Different Cultivars. Food Chem..

[80] Singh B.K., Bala M., Rai P.K. (2014). Fatty Acid Composition and Seed Meal Characteristics of Brassica and Allied Genera. Natl. Acad. Sci. Lett..

[81] Lee J.-E., Kim A.-J., Lee J.-E., Kim A.-J. (2020). Antioxidant Activity, Whitening and Anti-Wrinkle Effects of Leaf and Seed Extracts of *Brassica Juncea* L. Czern. Asian J. Beauty. Cosmetol..

[82] Jun H.-I., Wiesenborn D.P., Kim Y.-S. (2014). Antioxidant Activity of Phenolic Compounds from Canola (*Brassica Napus*) Seed. Food Sci. Biotechnol..

[83] Teh S.-S., Bekhit A.E.-D., Birch J. (2014). Antioxidative Polyphenols from Defatted Oilseed Cakes: Effect of Solvents. Antioxidants.

[84] Villaño D., López-Chillón M.T., Zafrilla P., Moreno D.A. (2019). Bioavailability of Broccoli Sprouts in Different Human Overweight Populations. J. Funct. Foods.

[85] Thomas M., Badr A., Desjardins Y., Gosselin A., Angers P. (2018). Characterization of Industrial Broccoli Discards (*Brassica Oleracea* Var. Italica) for Their Glucosinolate, Polyphenol and Flavonoid Contents Using UPLC MS/MS and Spectrophotometric Methods. Food Chem..

[86] Cartea M.E., Francisco M., Soengas P., Velasco P. (2010). Phenolic Compounds in Brassica Vegetables. Molecules.

[87] Vallejo F., Tomás-Barberán F.A., Ferreres F. (2004). Characterisation of Flavonols in Broccoli (*Brassica oleracea* L. Var. Italica) by Liquid Chromatography–UV Diode-Array Detection–Electrospray Ionisation Mass Spectrometry. J. Chromatogr. A.

[88] Price K.R., Casuscelli F., Colquhoun I.J., Rhodes M.J.C. (1997). Hydroxycinnamic Acid Esters from Broccoli Florets. Phytochemistry.

[89] Shao Y., Jiang J., Ran L., Lu C., Wei C., Wang Y. (2014). Analysis of Flavonoids and Hydroxycinnamic Acid Derivatives in Rapeseeds (*Brassica napus* L. Var. Napus) by HPLC-PDA–ESI (−)-MS n/HRMS. J. Agric. Food Chem..

[90] Auger B., Marnet N., Gautier V., Maia-Grondard A., Leprince F., Renard M., Guyot S., Nesi N., Routaboul J.-M. (2010). A Detailed Survey of Seed Coat Flavonoids in Developing Seeds of *Brassica napus* L. J. Agric. Food Chem..

[91] Blažević I., Montaut S., Burčul F., Olsen C.E., Burow M., Rollin P., Agerbirk N. (2020). Glucosinolate Structural Diversity, Identification, Chemical Synthesis and Metabolism in Plants. Phytochemistry.

[92] Pardini A., Tamasi G., De Rocco F., Bonechi C., Consumi M., Leone G., Magnani A., Rossi C. (2021). Kinetics of Glucosinolate Hydrolysis by Myrosinase in Brassicaceae Tissues: A High-Performance Liquid Chromatography Approach. Food Chem..

[93] Gu H., Mao X., Du M. (2022). Metabolism, Absorption, and Anti-Cancer Effects of Sulforaphane: An Update. Crit. Rev. Food Sci. Nutr..

[94] Eisenschmidt-Bönn D., Schneegans N., Backenköhler A., Wittstock U., Brandt W. (2019). Structural Diversification during Glucosinolate Breakdown: Mechanisms of Thiocyanate, Epithionitrile and Simple Nitrile Formation. Plant J..

[95] Van Eylen D., Hendrickx M., Van Loey A. (2006). Temperature and Pressure Stability of Mustard Seed (*Sinapis alba* L.) Myrosinase. Food Chem..

[96] Wang N., Shen L., Qiu S., Wang X., Wang K., Hao J., Xu M. (2010). Analysis of the Isothiocyanates Present in Three Chinese Brassica Vegetable Seeds and Their Potential Anticancer Bioactivities. Eur. Food Res. Technol..

[97] Bassan P., Bhushan S., Kaur T., Arora R., Arora S., Vig A.P. (2018). Extraction, Profiling and Bioactivity Analysis of Volatile Glucosinolates Present in Oil Extract of *Brassica juncea* Var. Raya. Physiol. Mol. Biol. Plants.

[98] Truscott R.J.W., Burke D.G., Minchinton I.R. (1982). The Characterisation of a Novel Hydroxindole Glucosinolate. Biochem. Biophys. Res. Commun..

[99] Devi J.R., Thangam E.B. (2012). Mechanisms of Anticancer Activity of Sulforaphane from Brassica Oleracea in HEp-2 Human Epithelial Carcinoma Cell Line. Asian Pac. J. Cancer Prev..

[100] Liang H., Li C., Yuan Q., Vriesekoop F. (2007). Separation and Purification of Sulforaphane from Broccoli Seeds by Solid Phase Extraction and Preparative High-Performance Liquid Chromatography. J. Agric. Food Chem..

[101] Liang H., Li C., Yuan Q., Vriesekoop F. (2008). Application of High-Speed Countercurrent Chromatography for the Isolation of Sulforaphane from Broccoli Seed Meal. J. Agric. Food Chem..

[102] Jing W., Zhao X., Liu A., Wei F., Ma S. (2022). Two New Nitrogenous Compounds from the Seeds of *Brassica napus*. Chem. Nat. Compd..

[103] Ferreres F., Sousa C., Valentão P., Seabra R.M., Pereira J.A., Andrade P.B. (2007). Tronchuda Cabbage (*Brassica oleracea* L. Var. Costata DC) Seeds: Phytochemical Characterization and Antioxidant Potential. Food Chem..

[104] Park S.-A., Chung I.-M., Ahmad A. (2012). Chemical Composition of the Essential Oil and Petroleum Ether Extract of *Brassica napus* Seeds. J. Essent. Oil Bear. Plants..

[105] Peng C., Zhao S.-Q., Zhang J., Huang G.-Y., Chen L.-Y., Zhao F.-Y. (2014). Chemical Composition, Antimicrobial Property and Microencapsulation of Mustard (*Sinapis alba*) Seed Essential Oil by Complex Coacervation. Food Chem..

[106] Grygier A. (2022). Mustard Seeds as a Bioactive Component of Food. Food Rev. Int..

[107] Rasera G.B., Hilkner M.H., de Castro R.J.S. (2020). Free and Insoluble-Bound Phenolics: How Does the Variation of These Compounds Affect the Antioxidant Properties of Mustard Grains during Germination?. Food Res. Int..

[108] Rasera G.B., Hilkner M.H., de Alencar S.M., de Castro R.J.S. (2019). Biologically Active Compounds from White and Black Mustard Grains: An Optimization Study for Recovery and Identification of Phenolic Antioxidants. Ind. Crops Prod..

[109] Altemimi A., Lakhssassi N., Baharlouei A., Watson D.G., Lightfoot D.A. (2017). Phytochemicals: Extraction, Isolation, and Identification of Bioactive Compounds from Plant Extracts. Plants.

[110] Verma A., Sharma A., Rai P.K. (2019). Impact of Soxhlet Extraction Method on Oil Yield and Antioxidant Potential of *Brassica juncea*. J. Pharmacogn. Phytochem..

[111] Almushayti A.Y., Brandt K., Carroll M.A., Scotter M.J. (2021). Current Analytical Methods for Determination of Glucosinolates in Vegetables and Human Tissues. J. Chromatogr. A.

[112] Cirilli R., Gallo F.R., Multari G., Palazzino G., Mustazza C., Panusa A. (2020). Study of Solvent Effect on the Stability of Isothiocyanate Iberin, a Breakdown Product of Glucoiberin. J. Food Compos. Anal..

[113] Rochfort S.J., Jones R. (2011). Glucosinolate Phytochemicals from Broccoli (*Brassica oleracea* L. Var. *Botrytis* L.) Seeds and Their Potential Health Effects. Nuts and Seeds in Health and Disease Prevention.

[114] Zhang C., Di H., Lin P., Wang Y., Li Z., Lai Y., Li H., Sun B., Zhang F. (2022). Genotypic Variation of Glucosinolates and Their Breakdown Products in Mustard (*Brassica juncea*) Seeds. Sci. Hortic..

[115] Franco P., Spinozzi S., Pagnotta E., Lazzeri L., Ugolini L., Camborata C., Roda A. (2016). Development of a Liquid Chromatography–Electrospray Ionization–Tandem Mass Spectrometry Method for the Simultaneous Analysis of Intact Glucosinolates and Isothiocyanates in Brassicaceae Seeds and Functional Foods. J. Chromatogr. A.

[116] Sun J., Zhang M., Chen P. (2016). GLS-Finder: A Platform for Fast Profiling of Glucosinolates in Brassica Vegetables. J. Agric. Food Chem..

[117] Olsen C.E., Huang X.-C., Hansen C.I.C., Cipollini D., Ørgaard M., Matthes A., Geu-Flores F., Koch M.A., Agerbirk N. (2016). Glucosinolate Diversity within a Phylogenetic Framework of the Tribe Cardamineae (*Brassicaceae*) Unraveled with HPLC-MS/MS and NMR-Based Analytical Distinction of 70 Desulfoglucosinolates. Phytochemistry.

[118] Fabre N., Bon M., Moulis C., Fouraste I., Stanislas E. (1997). Three Glucosinolates from Seeds of *Brassica juncea*. Phytochemistry.

[119] Nicácio A.E., Rodrigues C.A., Visentainer J.V., Maldaner L. (2021). Evaluation of the QuEChERS Method for the Determination of Phenolic Compounds in Yellow (*Brassica alba*), Brown (*Brassica juncea*), and Black (*Brassica nigra*) Mustard Seeds. Food Chem..

[120] Aziz S.S., El-Zayat M.M., El-Khateeb A.Y. (2020). Phytochemical Characterization, Antioxidant and Antimicrobial Activities of *Brassica juncea* (L.) Mustard Seeds Aqueous and Ethanolic Extracts. J. Plant Prod..

[121] Baumert A., Milkowski C., Schmidt J., Nimtz M., Wray V., Strack D. (2005). Formation of a Complex Pattern of Sinapate Esters in Brassica Napus Seeds, Catalyzed by Enzymes of a Serine Carboxypeptidase-like Acyltransferase Family?. Phytochemistry.

[122] Wang J., Yu H., Zhao Z., Sheng X., Shen Y., Gu H. (2019). Natural Variation of Glucosinolates and Their Breakdown Products in Broccoli (*Brassica oleracea* Var. Italica) Seeds. J. Agric. Food Chem..

[123] Arora S., Vig A.P. (2015). Inhibition of DNA Oxidative Damage and Antimutagenic Activity by Dichloromethane Extract of Brassica Rapa Var. Rapa L. Seeds. Ind. Crops Prod..

[124] Matsumoto T., Shimizu N., Shigemoto T., Itoh T., Iida T., Nishioka A. (1983). Isolation of 22-Dehydrocampesterol from the Seeds of Brassica Juncea. Phytochemistry.

[125] Valette L., Fernandez X., Poulain S., Lizzani-Cuvelier L., Loiseau A. (2006). Chemical Composition of the Volatile Extracts from *Brassica oleracea* L. Var. Botrytis ‘Romanesco’Cauliflower Seeds. Flavour Fragr. J..

[126] Shabana M.M., Fathy F.I., Salama M.M., Hashem M.M. (2013). Cytotoxic and Antioxidant Activities of the Volatile Constituents of Brassica Tournefortii Gouan: Growing in Egypt. Cancer. Sci. Res..

[127] Dua A., Chander S., Agrawal S., Mahajan R. (2014). Antioxidants from Defatted Indian Mustard (*Brassica juncea*) Protect Biomolecules against in Vitro Oxidation. Physiol. Mol. Biol. Plants.

[128] Mullen W., Marks S.C., Crozier A. (2007). Evaluation of Phenolic Compounds in Commercial Fruit Juices and Fruit Drinks. J. Agric. Food Chem..

[129] Shen S., Callaghan D., Juzwik C., Xiong H., Huang P., Zhang W. (2010). ABCG2 Reduces ROS-mediated Toxicity and Inflammation: A Potential Role in Alzheimer’s Disease. J. Neurochem..

[130] Podsędek A. (2007). Natural Antioxidants and Antioxidant Capacity of Brassica Vegetables: A Review. LWT Food Sci. Technol..

[131] Azeem M., Hanif M., Mahmood K., Ameer N., Chughtai F.R.S., Abid U. (2022). An Insight into Anticancer, Antioxidant, Antimicrobial, Antidiabetic and Anti-Inflammatory Effects of Quercetin: A Review. Polym. Bull..

[132] Bangar S.P., Chaudhary V., Sharma N., Bansal V., Ozogul F., Lorenzo J.M. (2022). Kaempferol: A Flavonoid with Wider Biological Activities and Its Applications. Crit. Rev. Food Sci. Nutr..

[133] Parikh H., Khanna A. (2014). Pharmacognosy and Phytochemical Analysis of Brassica Juncea Seeds. Pharmacogn. J..

[134] Chaudhary A., Choudhary S., Sharma U., Vig A.P., Arora S. (2016). In Vitro Evaluation of Brassica Sprouts for Its Antioxidant and Antiproliferative Potential. Indian J. Pharm. Sci..

[135] Bopitiya D., Hearn M.T.W., Zhang J., Bennett L.E. (2022). Demonstration of Anti-Oxidant Properties of Mustard Seed (*Brassica juncea*) Protein Isolate in Orange Juice. Food Chem..

[136] Choe U., Li Y., Gao B., Yu L.U., Wang T.T.Y., Sun J., Chen P., Liu J., Yu L. (2018). Chemical Compositions of Cold-Pressed Broccoli, Carrot, and Cucumber Seed Flours and Their in Vitro Gut Microbiota Modulatory, Anti-Inflammatory, and Free Radical Scavenging Properties. J. Agric. Food Chem..

[137] Asaad N.K., Razooqi Q.A. (2022). Protective Role of the Aqueous Extract of Brassica Nigra Seed against Cadmium Chloride Toxicity in Lung Tissue and Hematological Parameters of Female Rats. Mater. Today Proc..

[138] Yuan H., Zhu M., Guo W., Jin L., Chen W., Brunk U.T., Zhao M. (2011). Mustard Seeds (*Sinapis alba* Linn) Attenuate Azoxymethane-Induced Colon Carcinogenesis. Redox Report.

[139] Xu X., Dai M., Lao F., Chen F., Hu X., Liu Y., Wu J. (2020). Effect of Glucoraphanin from Broccoli Seeds on Lipid Levels and Gut Microbiota in High-Fat Diet-Fed Mice. J. Funct. Foods..

[140] Virk P., Alajmi S.T.A., Awad M., Elobeid M., Ortashi K.M.O., Asiri A.M., Merghani N.M., Fouad D. (2022). Attenuating Effect of Indian Mustard (*Brassica juncea*) Seed and Its Nano Formulation on Arsenic Induced-Oxidative Stress and Associated Genotoxicity in Rat. J. King Saud Univ. Sci..

[141] Chaudhary A., Sharma U., Vig A.P., Singh B., Arora S. (2014). Free Radical Scavenging, Antiproliferative Activities and Profiling of Variations in the Level of Phytochemicals in Different Parts of Broccoli (*Brassica oleracea italica*). Food Chem..

[142] Anwar F., Kalsoom U., Sultana B., Mushtaq M., Mehmood T., Arshad H.A. (2013). Effect of Drying Method and Extraction Solvent on the Total Phenolics and Antioxidant Activity of Cauliflower (*Brassica oleracea* L.) Extracts. Int. Food Res. J..

[143] Kwak Y., Lee J., Ju J. (2016). Anti-Cancer Activities of Brassica Juncea Leaves in Vitro. EXCLI J..

[144] Mori N., Shimazu T., Sasazuki S., Nozue M., Mutoh M., Sawada N., Iwasaki M., Yamaji T., Inoue M., Takachi R. (2017). Cruciferous Vegetable Intake Is Inversely Associated with Lung Cancer Risk among Current Nonsmoking Men in the Japan Public Health Center (JPHC) Study. J. Nutr..

[145] Fang Y., Yang C., Yu Z., Li X., Mu Q., Liao G., Yu B. (2021). Natural Products as LSD1 Inhibitors for Cancer Therapy. Acta Pharm. Sin. B.

[146] Ghanbari-Movahed M., Jackson G., Farzaei M.H., Bishayee A. (2021). A Systematic Review of the Preventive and Therapeutic Effects of Naringin against Human Malignancies. Front. Pharmacol..

[147] Ackland M.L., Van De Waarsenburg S., Jones R. (2005). Synergistic Antiproliferative Action of the Flavonols Quercetin and Kaempferol in Cultured Human Cancer Cell Lines. In Vivo.

[148] Almatroodi S.A., Alsahli M.A., Almatroudi A., Verma A.K., Aloliqi A., Allemailem K.S., Khan A.A., Rahmani A.H. (2021). Potential Therapeutic Targets of Quercetin, a Plant Flavonol, and Its Role in the Therapy of Various Types of Cancer through the Modulation of Various Cell Signaling Pathways. Molecules.

[149] Imani A., Maleki N., Bohlouli S., Kouhsoltani M., Sharifi S., Maleki Dizaj S. (2021). Molecular Mechanisms of Anticancer Effect of Rutin. Phytother. Res..

[150] Li S., Lin J., Wei J., Zhou L., Wang P., Qu S. (2022). Sinigrin Impedes the Breast Cancer Cell Growth through the Inhibition of PI3K/AKT/MTOR Phosphorylation-Mediated Cell Cycle Arrest. J. Environ. Pathol. Toxicol. Oncol..

[151] Jie M., Cheung W.M., Yu V., Zhou Y., Tong P.H., Ho J.W.S. (2014). Anti-Proliferative Activities of Sinigrin on Carcinogen-Induced Hepatotoxicity in Rats. PLoS ONE.

[152] Melim C., Lauro M.R., Pires I.M., Oliveira P.J., Cabral C. (2022). The Role of Glucosinolates from Cruciferous Vegetables (*Brassicaceae*) in Gastrointestinal Cancers: From Prevention to Therapeutics. Pharmaceutics.

[153] Ağagündüz D., Şahin T.Ö., Yılmaz B., Ekenci K.D., Duyar Özer Ş., Capasso R. (2022). Cruciferous Vegetables and Their Bioactive Metabolites: From Prevention to Novel Therapies of Colorectal Cancer. Evid. Based Complement. Altern. Med..

[154] Chang P., Tsai F., Bau D., Hsu Y., Yang J., Tu M., Chiang S. (2021). Potential Effects of Allyl Isothiocyanate on Inhibiting Cellular Proliferation and Inducing Apoptotic Pathway in Human Cisplatin-Resistant Oral Cancer Cells. J. Formos. Med. Assoc..

[155] Kaiser A.E., Baniasadi M., Giansiracusa D., Giansiracusa M., Garcia M., Fryda Z., Wong T.L., Bishayee A. (2021). Sulforaphane: A Broccoli Bioactive Phytocompound with Cancer Preventive Potential. Cancers.

[156] Dinh T.N., Parat M.-O., Ong Y.S., Khaw K.Y. (2021). Anticancer Activities of Dietary Benzyl Isothiocyanate: A Comprehensive Review. Pharmacol. Res..

[157] Li X., Zhao Z., Li M., Liu M., Bahena A., Zhang Y., Zhang Y., Nambiar C., Liu G. (2018). Sulforaphane Promotes Apoptosis, and Inhibits Proliferation and Self-Renewal of Nasopharyngeal Cancer Cells by Targeting STAT Signal through MiRNA-124-3p. Biomed. Pharmacother..

[158] Wang Y., Wu H., Dong N., Su X., Duan M., Wei Y., Wei J., Liu G., Peng Q., Zhao Y. (2021). Sulforaphane Induces S-Phase Arrest and Apoptosis via P53-Dependent Manner in Gastric Cancer Cells. Sci. Rep..

[159] El-Daly S.M., Gamal-Eldeen A.M., Gouhar S.A., Abo-Elfadl M.T., El-Saeed G. (2020). Modulatory Effect of Indoles on the Expression of MiRNAs Regulating G1/S Cell Cycle Phase in Breast Cancer Cells. Appl. Biochem. Biotechnol..

[160] Wang N., Wang W., Liu C., Jin J., Shao B., Shen L. (2016). Inhibition of Growth and Induction of Apoptosis in A549 Cells by Compounds from Oxheart Cabbage Extract. J. Sci. Food Agric..

[161] Ahmed A.G., Hussein U.K., Ahmed A.E., Kim K.M., Mahmoud H.M., Hammouda O., Jang K.Y., Bishayee A. (2020). Mustard Seed (Brassica Nigra) Extract Exhibits Antiproliferative Effect against Human Lung Cancer Cells through Differential Regulation of Apoptosis, Cell Cycle, Migration, and Invasion. Molecules.

[162] Kuwahara H., Kanazawa A., Wakamatu D., Morimura S., Kida K., Akaike T., Maeda H. (2004). Antioxidative and Antimutagenic Activities of 4-Vinyl-2, 6-Dimethoxyphenol (Canolol) Isolated from Canola Oil. J. Agric. Food Chem..

[163] Zhu M., Yuan H., Guo W., Li X., Jin L., Brunk U.T., Han J., Zhao M., Lu Y. (2012). Dietary Mustard Seeds (*Sinapis alba* Linn) Suppress 1, 2-Dimethylhydrazine-Induced Immuno-Imbalance and Colonic Carcinogenesis in Rats. Nutr. Cancer.

[164] Salman I.N., Hanna D.B., Mshimesh B.A.-R. (2022). Antiproliferative Activity of Brassica Nigra Seeds Extract in Liver Tissue of Mice Exposed to Phenobarbital. Al Mustansiriyah J. Pharm. Sci..

[165] Muluye A.B., Melese E., Adinew G.M. (2015). Antimalarial Activity of 80% Methanolic Extract of *Brassica nigra* (L.) Koch. (*Brassicaceae*) Seeds against Plasmodium Berghei Infection in Mice. BMC Complement. Altern. Med..

[166] Wang Y., Chang R.Y.K., Britton W.J., Chan H.-K. (2022). Advances in the Development of Antimicrobial Peptides and Proteins for Inhaled Therapy. Adv. Drug Deliv. Rev..

[167] Wang C., Zhang Y., Zhang W., Yuan S., Ng T., Ye X. (2019). Purification of an Antifungal Peptide from Seeds of *Brassica oleracea* Var. Gongylodes and Investigation of Its Antifungal Activity and Mechanism of Action. Molecules.

[168] Pacheco-Cano R.D., Salcedo-Hernández R., Casados-Vázquez L.E., Wrobel K., Bideshi D.K., Barboza-Corona J.E. (2020). Class I Defensins (BraDef) from Broccoli (*Brassica oleracea* Var. Italica) Seeds and Their Antimicrobial Activity. World J. Microbiol. Biotechnol..

[169] Thery T., Lynch K.M., Zannini E., Arendt E.K. (2020). Isolation, Characterisation and Application of a New Antifungal Protein from Broccoli Seeds–New Food Preservative with Great Potential. Food Control.

[170] Mignone G., Shwaiki L.N., Arendt E.K., Coffey A. (2022). Isolation of the Mustard Napin Protein Allergen Sin a 1 and Characterisation of Its Antifungal Activity. Biochem. Biophys. Rep..

[171] Khaliq B., Abdalla M., Mehmood S., Saeed A., Munawar A., Saeed M.Q., Saeed Q., Ibrahim M., Ali Z., Hussain S. (2022). Comprehensive Structural and Functional Characterization of a Seed γ-Thionin as a Potent Bioactive Molecule against Fungal Pathogens and Insect Pests. Curr. Med. Chem..

[172] Bellostas N., Casanova E., Garcia-Mina J.M., Hansen L.M., Jørgensen L.N., Kudsk P., Madsen P.H., Sørensen J.C., Sørensen H. Biological Activity of Glucosinolate Derived Compounds Isolated from Seed Meal of Brassica Crops and Evaluated as Plant and Food Protection Agents. Proceedings of the 12th International Rapeseed Congress.

[173] García-Saldaña J.S., Parra-Delgado J., Campas-Baypoli O.N., Sánchez-Machado D.I., Cantú-Soto E.U., López-Cervantes J. (2020). Changes in Growth Kinetics and Motility Characteristics of *Escherichia coli* in the Presence of Sulphoraphane Isolated from Broccoli Seed Meal. Int. J. Food Sci. Technol..

[174] Bousquet J., Le Moing V., Blain H., Czarlewski W., Zuberbier T., de la Torre R., Lozano N.P., Reynes J., Bedbrook A., Cristol J.-P. (2021). Efficacy of Broccoli and Glucoraphanin in COVID-19: From Hypothesis to Proof-of-Concept with Three Experimental Clinical Cases. World Allergy Organ. J..

[175] Grover J.K., Yadav S., Vats V. (2002). Hypoglycemic and Antihyperglycemic Effect of Brassica Juncea Diet and Their Effect on Hepatic Glycogen Content and the Key Enzymes of Carbohydrate Metabolism. Mol. Cell. Biochem..

[176] Grover J.K., Yadav S.P., Vats V. (2003). Effect of Feeding Murraya Koeingii and Brassica Juncea Diet Kidney Functions and Glucose Levels in Streptozotocin Diabetic Mice. J. Ethnopharmacol..

[177] Thirumalai T., Therasa S.V., Elumalai E.K., David E. (2011). Hypoglycemic Effect of *Brassica juncea* (Seeds) on Streptozotocin Induced Diabetic Male Albino Rat. Asian Pac. J. Trop. Biomed..

[178] Kumar M., Sharma S., Vasudeva N. (2013). In Vivo Assessment of Antihyperglycemic and Antioxidant Activity from Oil of Seeds of Brassica Nigra in Streptozotocin Induced Diabetic Rats. Adv. Pharm. Bull..

[179] Kay B.A., Trigatti K., MacNeil M.B., Klingel S.L., Repin N., Goff H.D., Wright A.J., Duncan A.M. (2017). Pudding Products Enriched with Yellow Mustard Mucilage, Fenugreek Gum or Flaxseed Mucilage and Matched for Simulated Intestinal Viscosity Significantly Reduce Postprandial Peak Glucose and Insulin in Adults at Risk for Type 2 Diabetes. J. Funct. Foods.

[180] Wu G.-X., Lin Y.-X., Ou M.-R., Tan D.-F. (2002). An Experimental Study (I) on the Inhibition of Prostatic Hyperplasia with Extract of Seeds of *Brassica alba*. Zhongguo Zhong Yao Za Zhi.

[181] Wu G.-X., Lin Y., Ou M.-R., Tan D. (2003). An Experimental Study (II) on the Inhibition of Prostatic Hyperplasia by Extract of Seeds of *Brassica alba*. Zhongguo Zhong Yao Za Zhi.

[182] Eckel R.H., Grundy S.M., Zimmet P.Z. (2005). The Metabolic Syndrome. Lancet.

[183] Chew S.C. (2020). Cold-Pressed Rapeseed (*Brassica napus*) Oil: Chemistry and Functionality. Food Res. Int..

[184] Palomäki A., Pohjantähti-Maaroos H., Wallenius M., Kankkunen P., Aro H., Husgafvel S., Pihlava J.-M., Oksanen K. (2010). Effects of Dietary Cold-Pressed Turnip Rapeseed Oil and Butter on Serum Lipids, Oxidized LDL and Arterial Elasticity in Men with Metabolic Syndrome. Lipids Health Dis..

[185] Chakraborty S., Paul K., Mallick P., Pradhan S., Das K., Chakrabarti S., Nandi D.K., Bhattacharjee P. (2019). Consortia of Bioactives in Supercritical Carbon Dioxide Extracts of Mustard and Small Cardamom Seeds Lower Serum Cholesterol Levels in Rats: New Leads for Hypocholesterolaemic Supplements from Spices. J. Nutr. Sci..

[186] Bhandari R., Kaur J., Kaur S., Kuhad A. (2021). The Nrf2 Pathway in Psychiatric Disorders: Pathophysiological Role and Potential Targeting. Expert Opin. Ther. Targets..

[187] Bent S., Lawton B., Warren T., Widjaja F., Dang K., Fahey J.W., Cornblatt B., Kinchen J.M., Delucchi K., Hendren R.L. (2018). Identification of Urinary Metabolites That Correlate with Clinical Improvements in Children with Autism Treated with Sulforaphane from Broccoli. Mol. Autism.

[188] Lietzow J. (2021). Biologically Active Compounds in Mustard Seeds: A Toxicological Perspective. Foods.

[189] Knutsen H.K., Alexander J., Barregård L., Bignami M., Brüschweiler B., Ceccatelli S., Dinovi M., Edler L., Grasl-Kraupp B., EFSA Panel on Contaminants in the Food Chain (CONTAM) (2016). Erucic Acid in Feed and Food. EFSA J..

[190] FSANZ (Australia New Zealand Food Standards Code) (2016). Maximum Levels of Contaminants and Natural Toxicants. 19.

[191] Russo M., Yan F., Stier A., Klasen L., Honermeier B. (2021). Erucic Acid Concentration of Rapeseed (*Brassica napus* L.) Oils on the German Food Retail Market. Food Sci. Nutr..

[192] Beszterda M., Nogala-Kałucka M. (2019). Current Research Developments on the Processing and Improvement of the Nutritional Quality of Rapeseed (*Brassica napus* L.). Eur. J. Lipid Sci. Technol..

[193] Lei W.U., JIA Y., Gang W.U., LU C. (2015). Molecular Evidence for Blocking Erucic Acid Synthesis in Rapeseed (*Brassica napus* L.) by a Two-Base-Pair Deletion in FAE1 (Fatty Acid Elongase 1). J. Integr. Agric..

[194] Chao H., Guo L., Zhao W., Li H., Li M. (2022). A Major Yellow-Seed QTL on Chromosome A09 Significantly Increases the Oil Content and Reduces the Fiber Content of Seed in Brassica Napus. Theor. Appl. Genet..

[195] Lang C., Wang F., Liu R., Zheng T., Hu Z., Wu X., Wu G. (2022). Genetic Regulation of Fatty Acid Biosynthesis in *Brassica napus* Seeds Based on FAE1 and FAD2 Genes. Mol. Plant Breed..

[196] Pałgan K., Żbikowska-Gotz M., Bartuzi Z. (2018). Dangerous Anaphylactic Reaction to Mustard. Arch. Med. Sci..

[197] Sikorska-Zimny K., Beneduce L. (2021). The Glucosinolates and Their Bioactive Derivatives in Brassica: A Review on Classification, Biosynthesis and Content in Plant Tissues, Fate during and after Processing, Effect on the Human Organism and Interaction with the Gut Microbiota. Crit. Rev. Food Sci. Nutr..

[198] Matthews Z.M., Parton K.H., Collett M.G. (2020). Investigating the Cause of Brassica-Associated Liver Disease (BALD) in Cattle: Progoitrin-Derived Nitrile Toxicosis in Rats. Toxicon X.

[199] Collett M.G., Stegelmeier B.L., Tapper B.A. (2014). Could Nitrile Derivatives of Turnip (*Brassica rapa*) Glucosinolates Be Hepato-or Cholangiotoxic in Cattle?. J. Agric. Food Chem..

[200] Griffiths D.W., Birch A.N.E., Hillman J.R. (1998). Antinutritional Compounds in the Brasi Analysis, Biosynthesis, Chemistry and Dietary Effects. J. Hortic. Sci. Biotechnol..

[201] Mithen R.F. (2001). Glucosinolates and Their Degradation Products. Adv. Bot. Res..

[202] Tripathi M.K., Mishra A.S. (2007). Glucosinolates in Animal Nutrition: A Review. Anim. Feed Sci. Technol..

[203] Hebert M., Mhemdi H., Vorobiev E. (2020). Selective and Eco-Friendly Recovery of Glucosinolates from Mustard Seeds (Brassica Juncea) Using Process Optimization and Innovative Pretreatment (High Voltage Electrical Discharges). Food Bioprod. Process..

[204] Chandra A.K. (2010). Goitrogen in Food: Cyanogenic and Flavonoids Containing Plant Foods in the Development of Goiter. Bioactive Foods in Promoting Health.

[205] Truong T., Baron-Dubourdieu D., Rougier Y., Guénel P. (2010). Role of Dietary Iodine and Cruciferous Vegetables in Thyroid Cancer: A Countrywide Case–Control Study in New Caledonia. Cancer Causes Control.

[206] EFSA (European Food Safety Authority) (2010). Scientific Opinion on the Safety of Allyl Isothiocyanate for the Proposed Uses as a Food Additive. EFSA J..

[207] Tan Z., Xie Z., Dai L., Zhang Y., Zhao H., Tang S., Wan L., Yao X., Guo L., Hong D. (2022). Genome-and Transcriptome-wide Association Studies Reveal the Genetic Basis and the Breeding History of Seed Glucosinolate Content in Brassica Napus. Plant Biotechnol. J..

